# Lipid phosphate phosphatase-2 promotes tumor growth through increased c-Myc expression

**DOI:** 10.7150/thno.66230

**Published:** 2022-07-18

**Authors:** Xiaoyun Tang, Christopher R. Cromwell, Rongzong Liu, Roseline Godbout, Basil P. Hubbard, Todd P.W. McMullen, David N. Brindley

**Affiliations:** 1 Department of Biochemistry and Cancer Research Institute of Northern Alberta, University of Alberta, Edmonton, T6G 2S2, Canada; 2 Department of Pharmacology, University of Alberta, Edmonton, T6G 2S2, Canada; 3 Department of Oncology, Cross Cancer Institute, University of Alberta, Edmonton, T6G 1Z2, Canada; 4 Department of Surgery, University of Alberta, Edmonton, Alberta, T6G 2R7, Canada

**Keywords:** breast cancer, CRISPR/Cas9, cell cycle, cell proliferation, tumor growth

## Abstract

LPP2 is one of three enzymes in the lipid phosphate phosphatase family (LPP1-3) that dephosphorylate extracellular and intracellular bioactive lipid phosphates and pyrophosphates. LPP2 increases cell growth and LPP2 expression is elevated in a variety of malignancies, implying that LPP2 is a pro-tumorigenic factor.

**Methods:** LPP2 expression in human breast tumors and normal breast tissue was measured by qPCR. To understand the role of LPP2, we knocked out its expression in multiple cell lines using CRISPR/Cas9. Cell proliferation and migration were compared between wild type and LPP2 knockout cells. Cell cycle was measured by flow cytometry, and cell cycle proteins were determined by western blotting. Effects of LPP2 on tumor growth were investigated using syngeneic and xenograft mouse breast cancer models.

**Results:** LPP2 mRNA levels were higher in ER/PR positive, ER/HER2 positive, and triple negative human breast tumors, relative to normal breast tissue. Higher levels of LPP2 in breast tumors, hepatocellular carcinoma, pancreatic adenocarcinoma, and melanomas were prognostic of poorer survival. LPP2 mRNA expression is also increased in Hs-578T, MDA-MB-231, MCF7 and MDA-MB-468 breast cancer cell lines, relative to non-malignant Hs-578Bst, MCF10A and MCF-12A cells. LPP2 knockout in breast cancer cells decreased cell growth by inhibiting G1/S transition, whereas, increasing LPP2 levels in Hs-578Bst and MCF10A cells promoted proliferation. The effects of LPP2 on cell cycle were associated with changes in cyclin A2, cyclin B1, and cell cycle inhibitors, p27 or p21. The level of c-Myc was downregulated by knocking out LPP2, and it was partly restored by re-expressing LPP2. The positive correlation between the expression of LPP2 and c-Myc exists in multiple cancer cell lines including breast, lung, upper aerodigestive tract and urinary tract cancer. LPP2 knockout in MDA-MB-231 or 4T1 cells suppressed tumor formation in mouse breast cancer models, and decreased the *in vivo* expression of Ki67 and c-Myc of the cancer cells.

**Conclusion:** Targeting LPP2 could provide a new strategy for decreasing c-Myc expression and tumor growth.

## Introduction

Lipid phosphate phosphatases (LPP) belong to a phosphatase/phosphotransferase super family 1, 2. There are three isoforms of LPP: LPP1 (*PLPP1*), -2 (*PLPP2*) and -3 (*PLPP3*), which are located on the plasma membrane and internal membranes including endoplasmic reticulum and Golgi network 1-3. The catalytic sites of LPPs in the plasma membrane are on the outer side of the cell 4. This provides the “ecto-activity” of the LPPs, which degrade extracellular substrates such as lysophosphatidate (LPA) and sphingosine 1-phosphate (S1P) 1. Importantly, LPA and S1P are potent growth factors that increase inflammation and tumor growth 5-8 though different families of G protein coupled receptors.

In addition to attenuating signaling by extracellular LPA and S1P, the LPPs also dephosphorylate a broad range of other bioactive lipid phosphates and pyrophosphates including phosphatidate, ceramide 1-phosphate and diacylglycerol pyrophosphate 1-3. Regulation of these intracellular targets could act as a mechanism to regulate cell signaling downstream of the receptors 1, 3. For example, LPP1 decreases the activation of phospholipase D 9 and Ca^2+^-transients 10 induced by wls-31, which is a phosphonate analogue of LPA that activates LPA_1/2_ receptors. Wls-31 cannot be dephosphorylated by the LPPs and therefore it was concluded that the effect of LPP1 occurred by degrading a lipid phosphate/pyrophosphate formed downstream of receptor activation. Previous work showed that thrombin-induced ERK phosphorylation is also inhibited by LPP1 expression 11 and we demonstrated that LPP1 expression attenuated the activation of Ca^2+^-transients by the protease-activated receptor-1 (PAR1) in MDA-MB-231 breast cancer cells 10. These combined effects indicate a role for LPP1 in attenuating signaling by several classes of G-protein coupled receptors.

All of the LPP isoforms can dephosphorylate a wide variety of lipid phosphates when assayed *in vitro*, but the LPPs display greater substrate specificity *in vivo.* This could also be influenced by the abilities of the LPPs to access the various substrates in different locations in the cell 3. Although the LPPs may have some functional redundancy, there is substantial evidence that they have distinct biological functions. For example, LPP3 knockout (KO) in mice results in embryonic lethality 12, whereas mice with LPP2 KO or hypomorphs for LPP1 are viable 13, 14. Overexpression of Wunen (a homologue of human LPPs with highly conserved phosphatase domains) in *Drosophila* caused aberrant migration of primordial germ cells. This phenotype was mimicked by expression of mammalian LPP3, but not LPP1 15. While these studies help to reveal specific roles for different LPP isoforms in regulating cell functions, the exact mechanisms for these differences remain largely unknown.

The effects of LPP1 and LPP3 have been investigated in cancers since their expression is decreased in many cancers, including breast cancer 16-18. This accounts for the increase of LPA concentrations in the tumors 19-21. Increasing the low levels of LPP1 or LPP3 in cancer cells suppresses tumor growth and metastasis in breast and ovarian cancer models, respectively 10, 14. Low expression of LPP1 in breast cancer cells increases the levels of cyclin D1, D3 and matrix metalloproteinases through enhanced transcription by AP-1 involving cFos and cJun, which increases cell division 22. In breast cancer patients, higher LPP1 expression in tumors was associated with greater survival 22, whereas LPP3 levels did not show a prognostic association 22. In contrast to LPP1 and LPP3, LPP2 expression is upregulated in many cancers 23. Increasing LPP2 in fibroblasts promotes cell growth 24. A genomic screen between normal and transformed mesenchymal stem cells indicated that LPP2 expression is elevated in several cancer cell lines including MCF7, SK-LMS1, MG63 and U2OS 25. Knockdown of LPP2 suppressed anchorage-dependent cell growth in this study 25. Collectively, these studies demonstrate that LPP2 stimulates cell growth, in contrast to LPP1 and LPP3. Apart from this work, little is known about the role of LPP2 in regulating cell division and tumor growth. Therefore, we sought to uncover how LPP2 activity controls the growth of breast cancer cells and tumors.

We first demonstrated that LPP2 expression is increased in tumors from six types of human breast cancer relative to normal breast tissue, and that patients with the higher levels of LPP2 expression have poorer survival. LPP2 KO in breast cancer cells decreases the nuclear expression of c-Myc and inhibits the G1/S transition of the cell cycle. Moreover, we observed a positive correlation between LPP2 expression and c-Myc in 56 breast cancer cell lines. LPP2 KO in MDA-MB-231 cells significantly decreases breast tumor growth and lung metastasis in a mouse xenograft model. LPP2 KO also inhibits 4T1 breast tumor growth in a mouse syngeneic model. This study provides unique evidence that LPP2 is a potential therapeutic target for decreasing c-Myc expression and tumor growth in breast and possibly other tumors.

## Methods

### Cell lines and reagents

Breast cancer cell lines: MCF7, MDA-MB-231, MDA-MB-468, Hs-578T, 4T1, and non-transformed MCF10A, MCF-12A, Hs-578Bst, HEK293 cells were from ATCC (Manassas, VA). Matrigel^TM^ (354230) was from Corning (Corning, NY). Mycoplasma infection was excluded by testing the culture media with a PCR mycoplasma detection kit (G238, Applied Biological Materials Inc, Richmond, BC, Canada). LR clonase enzyme mix (11791019) was from ThermoFisher Scientific (Grand Island, NY). The transfection reagent PolyJet (SL100688) was from SignaGen Laboratories (Gaithersburg, MD). PfuUltra DNA polymerase (600385) was from Agilent Technologies (Santa Clara, CA). The UltRNA column purification kit (G487), reverse transcription master mix (G490) and EvaGreen qPCR MasterMix (MasterMix-ER) were from Applied Biological Materials Inc. (Richmond, BC, Canada). Guide RNA (gRNA) expression vector MLM3636 (43860), Cas9 expression vector JDS246 (43861), pENTR-GFP-N2 (19364), pLenti-PGK-Neo-DEST (19067), pMD2.G (12259), pRSV-Rev (12253) and pMDLg/pRRE (12251) were from Addgene (Cambridge, MA). Cas9 Nuclease, *S. pyogenes* (M0386T) was purchased from New England BioLabs (Ipswich, MA). Alt-R CRISPR-Cas9 crRNA and Alt-R CRISPR-Cas9 tracrRNA, ATTO 550 (1075927) were purchased from Integrated DNA Technologies (Coralville, IA). Rabbit anti-cyclin E (07-687) antibody, lovastatin (1370600) and propidium iodide (P4170) were from Millipore Sigma (Burlington, MA). Rabbit anti-c-Myc (5605), rabbit anti-phospho(S62)-c-Myc (13748), mouse anti-cyclin A2 (4656), rabbit anti-cyclin B1 (4138), rabbit anti-cyclin D1 (2978), mouse anti-cyclin D3 (2936), rabbit anti-p21 (2947), rabbit-anti p27 (2552), rabbit anti-Ki67 (9027), and rabbit anti-cleaved caspase 3 (9661) antibodies were from Cell Signaling Technology (Danvers, MA). Rabbit anti-phospho(T58)-c-myc (ab185655) and rabbit anti-c-Myc (ab32072) antibodies were from Abcam Inc. (Toronto, ON, Canada). Rabbit anti-cyclin D2 (C-17) antibodies were from Santa Cruz (Dallas, TX). Rabbit anti-GFP antibody was kindly provided by Dr. Luc Berthiaume (Department of Cell Biology, University of Alberta, Edmonton, Canada).

### Patient and cell line data analysis

Tumor samples were taken from breast cancer patients undergoing surgery at the University of Alberta Hospital, Edmonton, AB. Normal breast tissues used for the controls were taken from patients receiving breast reduction surgery. The Ethics Committee of the University of Alberta approved this investigation.

Microarray data and clinical information of breast cancer patient cohort were obtained from The Cancer Genome Atlas (TCGA) 26 or the Cancer Cell Line Encyclopedia (CCLE) of the Broad Institute and Novartis 27 at the website of cBioportal (www.cbioportal.org). Patients were stratified as high expression and low expression of LPP2 based on the Z score of the mRNA levels of LPP2 (Z < 0, low; Z > 0, high). Survival analysis and bivariate correlation of gene expression were performed.

### Generation of LPP2 knockout cell lines

Guide RNAs (gRNA) targeting human or mouse LPP2 were prepared as described previously 28 using Alt-R CRISPR-Cas9 tracrRNA, ATTO 550 (IDT) and Alt-R CRISPR-Cas9 crRNA (IDT). Assembled Cas9 ribonucleoprotein (RNP) complexes were transfected into HEK293 cells using Polyjet, and into MCF7, MDA-MB-231, and 4T1 cells using Lipofectamine CRISPRMAX according to the manufactures' instructions. Twenty-four h after transfection, positive cells were sorted on a BD FACSAria III sorting instrument by the Flow Cytometry Core at the University of Alberta into the wells of a 96-well plate at a density of one cell per well. Upon expansion of single colonies, approximately 1x10^5^ cells were lysed in DirectPCR Lysis Reagent (Viagen Biotech, Inc.) overnight at 55°C, with the remaining cells transferred to a 24-well plate. The following day, Proteinase K was heat inactivated by incubation at 85°C for 45 min. 1 µL of crude lysate was used as template for PCR amplification of the Cas9 target site using the following primer sets: 5ʹ-GGCCTTCTTCAGCTCCCATT-3ʹ and 5ʹ-GGCCACCGTCATCCTTGTAA-3ʹ for human cell lines, and 5ʹ-CATCACAGCTACTGTCATCCTT-3ʹ and 5ʹ-GAACCCTGAGACTGTCCATTT-3ʹ for the mouse cell line. The resulting PCR product was analyzed via Sanger sequencing to confirm the presence of Cas9-mediated gene disruption. LPP2 knockout clones were mixed for use in functional assays and animal models.

### LPP activity assays

Total LPP activity against [^3^H]labeled phosphatidic acid was measured as described by Jasinska et al. 4.

### Constructs for expression of LPP2

Human LPP2 was cloned from cDNA of HEK293 cells using primers 5'-CCAAGCTTACACCATGCAGCGGAGGTGGGTCTT-3' and 5'-TCCCCGCGGTAGGAGGAGGAGTGCGGGTATCC-3'. The LPP2 fragment was ligated into the pENTR1A-GFP-N2 vector using HindIII and SacII restriction sites, and then transferred into pLenti-PGK-Neo-DEST vector by LR recombination. Lentivirus was generated as described previously 10. Lentivirus coding GFP was generated follow the same procedure as LPP2 and used as a control virus. Cells were transduced with lentivirus and selected using G418 to establish stable cell lines.

### Cell proliferation assay in two-dimensional and three-dimensional culture

MCF7, MDA-MB-231, and HEK 293 cells were cultured in DMEM media containing 10% FBS. MCF10A cells were cultured in DMEM/F12 media supplemented with 10% FBS, 20 ng/ml EGF, 0.5 μg/ml hydrocortisone, 100 ng/ml cholera toxin, and 10 μg/ml insulin. Hs-578Bst cells were cultured in Hybri-care media (ATCC) supplemented with 10% FBS and 30 ng/ml EGF. To determine cell proliferation in 2-D culture, MCF7, MDA-MB-231, MCF10A, and HEK293 cells were seeded in 12-well plates (50,000 cells per well) and cell growth was monitored for 5 days. He-578Bst cells were seeded in 24-well plates (10,000 cells per well), and cell growth was monitored for 11 days. Cells were fixed with 4% paraformaldehyde and stained with 0.1% crystal violet dissolved in 10% methanol. Cells were washed 3 times with PBS. Crystal violet bound to cells was extracted with 10% acetic acid and OD_590nm_ was measured. For the 3-D culture, MCF7 cells were resuspended in DMEM (1.5 x 10^4^ cells/ml) supplemented with 2% growth factor-reduced Matrigel and 10% FBS. Cell suspensions (400 μl/well) were put onto the top of a thin layer of Matrigel (150 μl/well) in 8-well chamber slides (177402, Thermo Scientific). Cells were grown for 10 days, and fixed with 4% paraformaldehyde. Images were taken with AMG EVOS microscope (Electron Microscopy Sciences, PA) and the average size of cell colonies was measured by ImageJ software.

### Cell cycle measurement

Cells were cultured in 10-cm dishes to 80-90% confluency and then trypsinized and washed with PBS. Subsequently, cells were fixed with cold 70% ethanol and kept at 4^ o^C for 16 h and then washed with PBS and stained with propidium iodide (PI) (20 μg/ml PI, 100 μg/ml RNase A in PBS). The cell cycle was analyzed by flow-cytometry (BD FACSCanto II, BD Biosciences).

To measure the G1/S transition, MDA-MB-231 cells were blocked at G1 phase by treating with 10 μM Lovastatin for 36 h. Cells were then cultured in Lovastatin-free medium for another 8 and 24 h followed by cell cycle measurement.

To measure G1/S transition without artificial drug-induced synchronization, cells were pulse labeled with 10 μM BrdU in DMEM/10% FBS for 1 h to label cells in S-phase and then cultured in fresh DMEM/10% medium for another 3, 14 and 23 h. Cells were trypsinized and fixed with cold 70% ethanol and kept in 4^ o^C for 16 h and washed with PBS. Cells were treated with 2 M HCl for 30 min and washed with PBS. Cells were stained with FITC-conjugated mouse anti-BrdU antibody for 1 h and washed with PBS. Cells were then stained with PI and analyzed by flow-cytometry.

### Nuclear fractionation

Cells in 6-cm dishes were washed twice with ice-cold PBS followed by adding 0.5 ml of lysis buffer: 10 mM HEPES; pH 7.5, 10 mM KCl, 0.1 mM EDTA, 1 mM dithiothreitol (DTT), 0.5% Nonidet-40 and protease inhibitors. Cells were collected by scraping and kept on ice for 30 min. After centrifugation at 1,500 g for 5 min, the nuclear pellets were washed three times with lysis buffer, and then sonicated in RIPA buffer. The supernatant was collected by centrifugation at 12,000 g for 15 min at 4°C as nuclear extract.

### Real-time PCR, PCR array and Western blotting

mRNA levels were determined by qRT-PCR using glyceraldehyde 3-phosphate dehydrogenase (GAPDH) as reference mRNA 10. Gene expression related to cancer signaling pathways and cancer stem cells was measured using the RT^2^ Profiler PCR Array (PAHS-033Z and PAFD-176Z, QIAGEN) according to the manufacturer's instructions. Protein levels were measured by Western blotting as described previously 10. Immunoblots were analyzed using an Odyssey infrared imaging system (LI-COR Biosciences, NE).

### Cell migration assay

Cell migration assays were performed using a 96-well Boyden chamber (MBB96, Neuro Probe) as described previously 10.

### Mouse breast tumor models

Syngeneic and xenograft orthotopic mouse breast cancer models were established by inoculating 4T1 or MDA-MB-231 cells into the mammary fat pads of female BALB/c or NSG (NOD scid gamma) mice, respectively, as reported previously 10. An experimental metastasis model was established by tail vein injection of MDA-MB-231 cells in NSG mice 10. All procedures were performed in accordance with the Canadian Council of Animal Care as approved by the University of Alberta Animal Welfare Committee. Tumor growth was monitored by two orthogonal caliper measurements and tumor volume was estimated using the equation width^2^ x length/2.

### Immunohistochemistry

Tissues were fixed with 10% formalin followed with paraffin embedding and sectioning. Sample treatment and immunostaining were performed according to the standard procedure using the HRP/DAB detection IHC kit (ab64261) from Abcam Inc (Toronto, ON, Canada). Heating with citrate buffer, pH 6.0, in a pressure cooker was used for antigen retrieval. Positive staining events for Ki67, c-Myc, and cleaved caspase 3 were analyzed by ImageJ software. The average results of 5 fields were calculated for each sample.

### Statistical analysis

Survival curves are estimated with the Kaplan-Meier method and compared statistically with the log rank test using MedCalc (Version 14.12.0). Bivariate correlation of gene expression levels was determined by Pearson correlation coefficient. Other results were analyzed by a student t-test or ANOVA followed by Tukey or Bonferroni test. *P* < 0.05 was considered statistically significant.

## Results

### Elevation of LPP2 mRNA levels in human breast tumors correlates with poor survival prognosis in breast cancer patients

**Figure 1:**
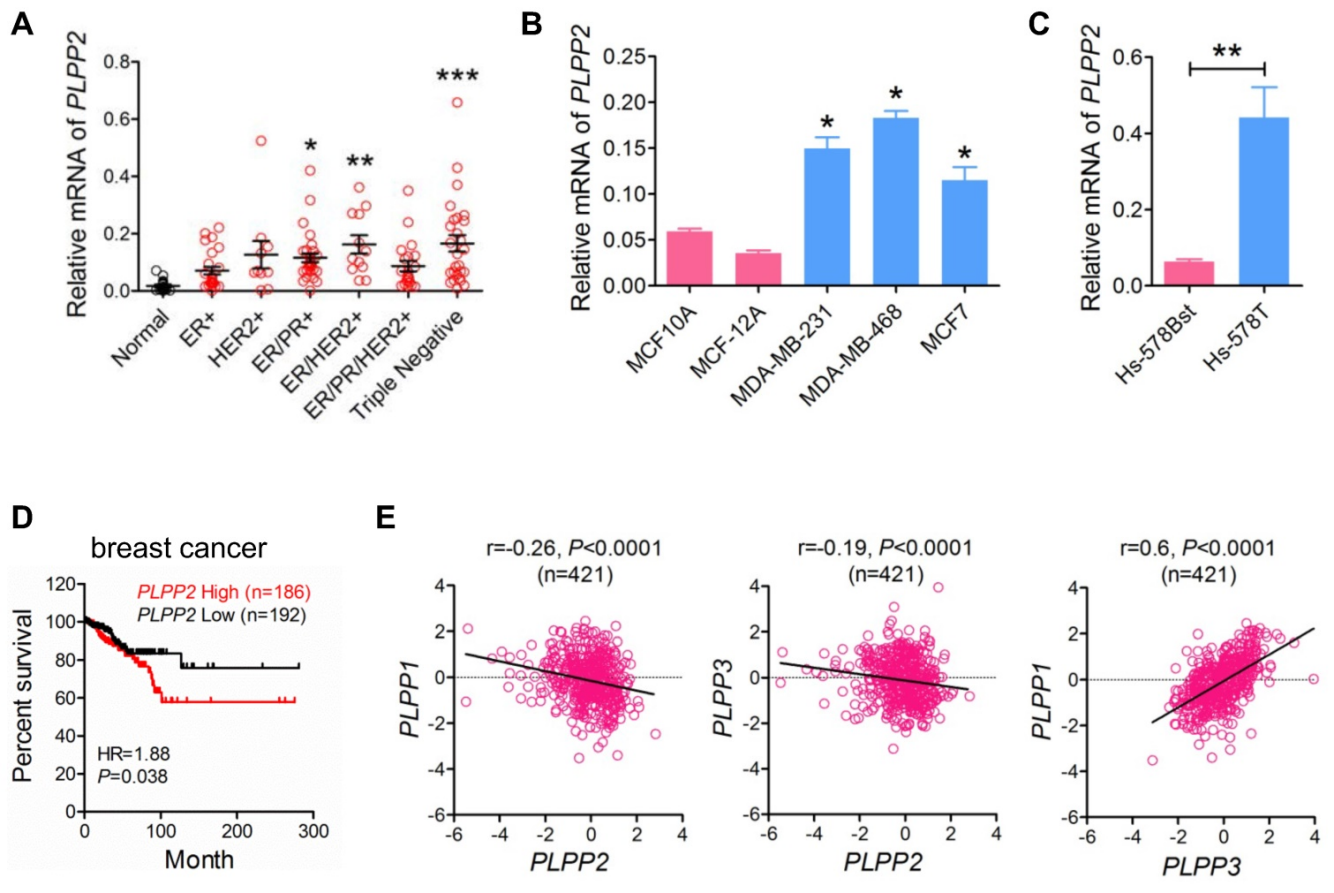
** LPP2 expression is increased in human breast tumors and shows a poor prognostic association with disease-free survival in patients. A:** Breast tumor samples were characterized as ER+ (n = 25), HER2+ (n = 10), ER/PR+ (n = 31), ER/HER2+ (n = 12), ER/PR/HER2+ (n = 21), and triple-negative (n = 27) and mRNA levels of LPP2 are shown relative to normal breast tissue (n = 18). **B:** LPP2 (*PLPP2*) expression was significantly higher in MDA-MB-231, MDA-MB-468 and MCF7 breast cancer cells relative to non-transformed MCF10A and MCF12A cells. **C:** LPP2 (*PLPP2*) expression was significantly higher in Hs- 578T breast cancer cells than in the patient-matching Hs-578Bst fibroblasts. **D:** Disease-free survival curves from breast cancer patients with high (Z score > 0) and low (Z score < 0) mRNA levels of LPP2 (*PLPP2*) were plotted using data extracted from TCGA database. Results were analyzed by log rank test. **E:** LPP1 (*PLPP1*) and LPP3 (*PLPP3*) expression showed a negative correlation with LPP2 (*PLPP2*), LPP1 and LPP3 expression showed a positive correlation. Correlation was determined with Pearson correlation coefficient. Results were analyzed by two tail t-test or ANOVA followed with Tukey test. *P < 0.05, **P < 0.01, ***P < 0.0001 compared with control.

We measured the mRNA levels of LPPs in a series of human breast tumors collected from patients in the Edmonton region. LPP2 mRNA (*PLPP2*) was 5.4, 8.1, and 8.2-fold higher in ER/PR positive, ER/HER2 positive, and triple negative breast cancer specimens, respectively, compared to normal breast tissue. There were also apparent increases in LPP2 mRNA in ER positive (3.0-fold), HER2 positive (6.0-fold), and ER/PR/HER2 positive (3.8-fold) breast cancer compared to normal breast tissue, although these did not reach statistical significance (Figure 1A). When all breast cancer samples were combined together, there was a significant 6.6-fold increase in LPP2 mRNA expression compared to normal breast tissue (*P* = 0.0002, results not shown). The human breast cancer cell lines MCF7, MDA-MB-231, and MDA-MB-468 also expressed significantly higher levels of LPP2 than non-transformed MCF10A and MCF-12A mammary epithelial cells (Figure 1B). Hs-578T breast cancer cells and Hs-578Bst mammary fibroblasts are paired cell lines that originated from the same patient 29. LPP2 mRNA was 6.9-fold higher in Hs-578T cells relative to Hs-578Bst cells (Figure 1C).

We analyzed the data from TCGA that contains 817 breast cancer patients 26. The patients were stratified as high (Z score > 0, n = 186) and low (Z score < 0, n = 192) expression of LPP2. High expression of LPP2 in breast tumors was significantly associated with the lower survival rates (Figure 1D). A similar correlation was also obtained for human hepatocellular cancer, pancreatic cancer, and melanoma (Supplementary Figure 1A-C), but was not as strong as in breast cancer (Hazard Ratio (HR) =1.88 in breast cancer *vs* 1.52 in hepatocellular cancer, 1.59 in pancreatic cancer, and 1.57 in melanoma). This suggested that the effect of LPP2 is probably affected by other factors in tumor progression and might be cancer type dependent. Four hundred and twenty one patients from this database had complete mRNA data for LPP1, 2, and 3, LPP2 expression in this cohort manifested a weak but significant negative correlation with LPP1 and LPP3, whereas the expression of LPP1 showed a significant positive correlation with LPP3 (Figure 1E).

These results establish that LPP2 levels are increased in several human tumors and that higher LPP2 expression is prognostic of poor survival.

### LPP2 knockout inhibits cell growth but not migration

We then inactivated the LPP2 gene to understand the consequences of high LPP2 expression in cancer cells. Since we were unable to obtain a specific band for endogenous LPP2 protein using any of the commercial antibodies that we tested (not shown), we use alternative approaches to validate KO of the LPP2 gene. We employed targeted amplification primers (5ʹ-CTTCCTAGCCGTCTGCGACC-3ʹ and 5ʹ-TGCCCACTTCCAACAGAGTC-3ʹ) designed to be complementary to the sequence being targeted for cleavage by Cas9, which is in exon 3 of the human LPP2 gene. Insertion or deletion (indels) of nucleotides caused by non-homologous end joining (NHEJ) repair following Cas9 cleavage of this site results in sequence changes that prevent binding of the targeted primer. No PCR products were amplified from the cDNA of MCF7 cells with LPP2 KO using these primers (Supplementary Figure 2A). Genomic sequencing was also used to confirm indels in the LPP2 gene (Supplementary Figure 2B). Total LPP activity was decreased by ~75%, 27%, and 19% when LPP2 was deleted from the MCF-7, MDA-MB-231, and HEK293 cells, respectively (Supplementary Figure 2C). The residual LPP activity likely arises from LPP1 and LPP3, for which mRNA levels were not affected by depletion of LPP2 (Supplementary Figure 2D, E).

**Figure 2:**
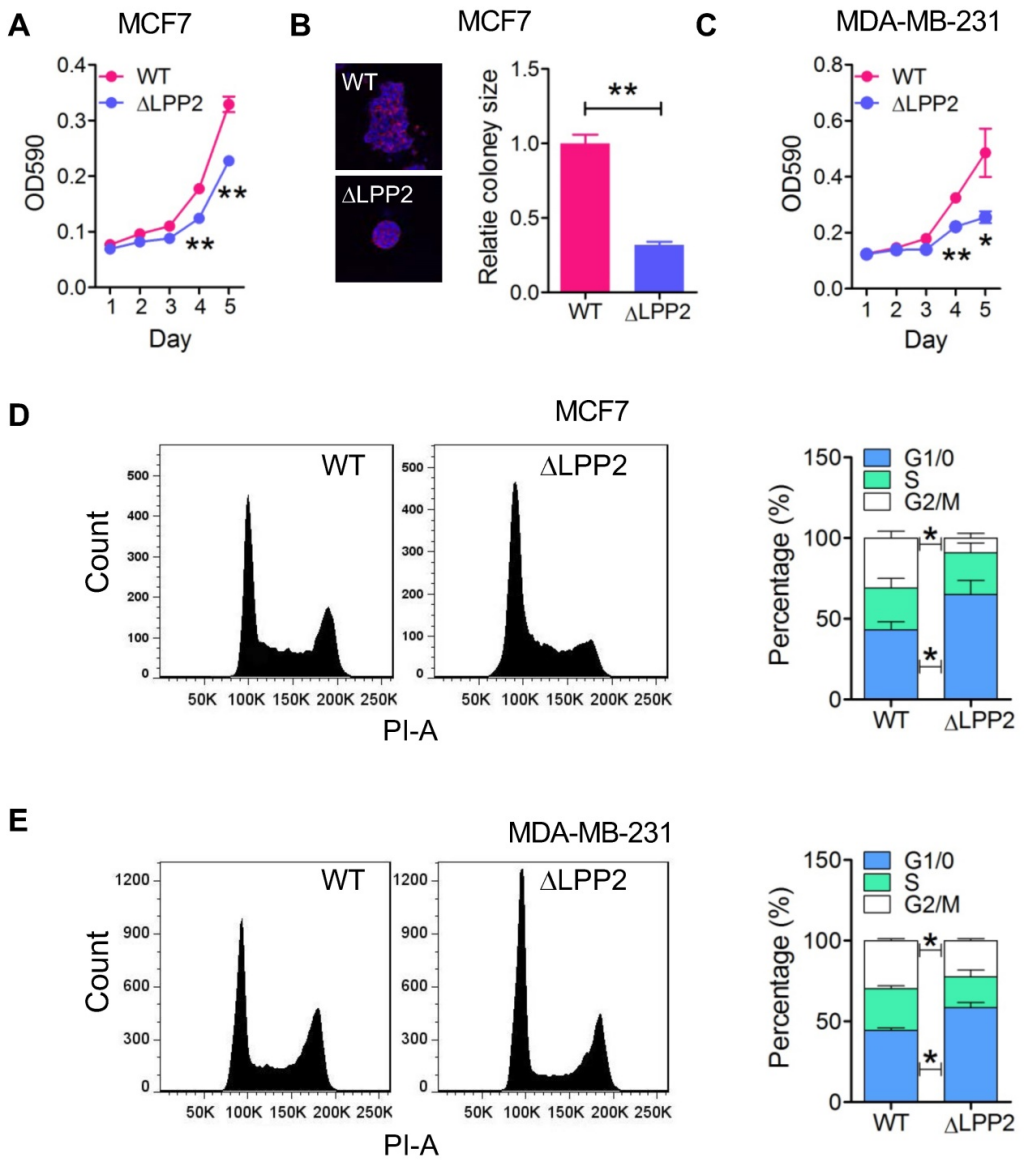
** Knockout of LPP2 (ΔLPP2) inhibits cell growth. A and B:** MCF7 cell growth in 2D and 3D culture were inhibited by knockout of LPP2. Images are the wild type (WT) and ΔLPP2 colonies stained with DAPI (blue) and phalloidin (red) in the same scale. **C:** Knockout of LPP2 inhibited proliferation of MDA-MB- 231 cells. **D and E:** Knockout of LPP2 increased the percentage of MCF7 and MDA-MB-231 cells in the G1/0 phase, and decreased cells in the G2/M phase of the cell cycle. Results are means ± SE from three experiments per group and analyzed by ANOVA followed with Tukey test. *P < 0.05, **P < 0.01 compared with WT.

LPP2 KO significantly inhibited the growth of MCF7 and MDA-MB-231 cells in monolayer culture by ~31% and 47% respectively (Figure 2A and C) relative to the wild type cells. The inhibition was more potent in 3-D culture where there was a 68% decrease in colony size of LPP2 KO MCF7 cells (Figure 2B). Similarly, LPP2 KO inhibited the growth of non-transformed HEK293 cells (Supplementary Figure 3A). LPP2 KO did not alter the cleavage of PARP, caspase-3 or caspase-9, and it did not affect the level of bcl-2 in MCF7 and MDA-MB-231 cells (Supplementary Figure 4A), suggesting that the inhibition of cell proliferation was not related to increased apoptosis. The response to stimulation by IGF, EGF and LPA was investigated in both wild type and LPP2 KO MDA-MB-231 cells by measuring Akt and ERK phosphorylation. There were no significant changes in IGF-, EGF- and LPA-induced Akt and ERK phosphorylation (Supplementary Figure 4B), indicating that LPP2 KO does not appear to affect the receptors for these agonists or their downstream signaling. Similar results were also shown in HEK293 cells (Supplementary Figure 4C and D).

We next determined if the inhibition of cell growth by LPP2 KO was reflected by changes in the cell cycle. LPP2 KO in MCF7 and MDA-MB-231 cells significantly increased the percentage of cells in G1/0 phase and decreased the percentage of cells in the G2/M phase (Figure 2D and E), demonstrating that LPP2 KO promotes a partial G1/0 phase arrest.

We also tested if LPP2 KO could affect cell migration since this is an important aspect of cancer metastasis. LPP2 KO in MDA-MB-231 cells did not alter LPA- or EGF-induced migration in a Boyden chamber assay (Supplementary Figure 5A and B). These results indicate that the major effect of LPP2 on cancer progression likely stems from regulation of the cell cycle. Therefore, we focused on characterizing the effects of LPP2 KO on G1/S transition.

### LPP2 knockout inhibits G1/S transition

**Figure 3:**
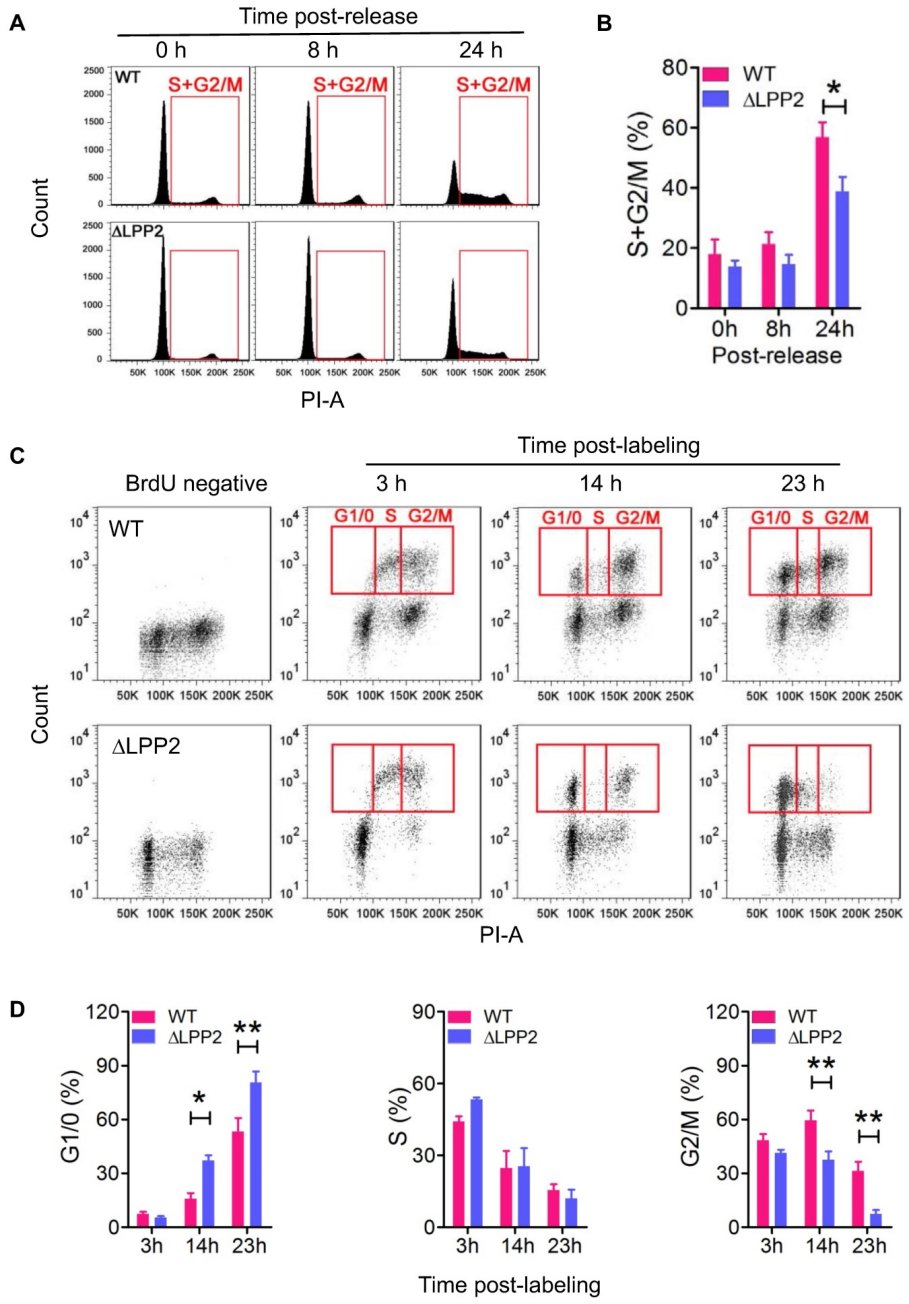
** Knockout of LPP2 (ΔLPP2) caused cell cycle block in G1/0. A and B:** MDA-MB-231 cells were synchronized in G1/0 phase by 10 µM Lovastatin for 36 h and then released. Cells that entered S and G2/M phases (indicated by red squares) were measured over time. LPP2 KO significantly decreased the percentage of cells in S and G2/M phase at 24 h after releasing compared with the wild type (WT) cells. **C and D:** MCF-7 cells were pulse labeled with BrdU for 1 h and stained with PI. The BrdU-positive cells in G1/0, S and G2/M phases (indicated by red squares) were measured over time. LPP2 KO significantly increased cells in G1/0 phase and decreased cells in G2/M phase at 14 and 23 h after labeling compared with the wild type (WT) cells. Results are means ± SE from three experiments per group and analyzed by ANOVA followed with Bonferroni test. *P < 0.05, **P < 0.01.

MDA-MB-231 cells were treated with Lovastatin to synchronize cells in the G0/1 phase 30. The cells were then released from this arrest by replacing with fresh Lovastatin-free medium. Cell re-entry into S and G2/M phases was measured by flow-cytometry. Treatment with 10 μM Lovastatin for 36 h arrested more than 80% of the cells in the G1/0 phase. After the release, LPP2 KO cells showed significantly slower re-entry into the S and G2/M phases relative to wild type cells (Figure 3A, B).

We also used BrdU labeling to measure dynamic changes in the cell cycle to exclude the possibility that using artificial synchronization could affect cell viability and produce a false-positive result. To do this, MCF7 cells were pulsed with BrdU for 1 h to label cells in S phase. We then monitored the dynamic change of the BrdU-positive cells in the cell cycle by co-staining with PI (Figure 3C). The majority of the BrdU-positive wild type and LPP2 KO cells were in the S and G2/M phases at 3 h after BrdU labeling of the cells. After 14 and 23 h, the BrdU-positive cells had divided to re-enter the G1/0 phase. The percentage of LPP2 KO cells in the G1/0 phase was 2.3-fold and 1.5-fold higher, respectively, at 14 and 23 h than wild type cells. This indicated an accumulation of LPP2 KO cells in the G1/0 phase, whereas the wild type cells progressed more rapidly through the cell cycle. This was compatible with the increase of 1.5-fold and 4.3-fold in the percentage of wild type cells compared to LPP2 KO cells that were present in the subsequent G2/M phase at 14 and 23 h (Figure 3D). These results establish that the cell cycle changes are due to LPP2 KO, and that they were not the consequence of heterogeneous cell synchronization.

### LPP2 knockout changes the expression of cell cycle regulators

**Figure 4:**
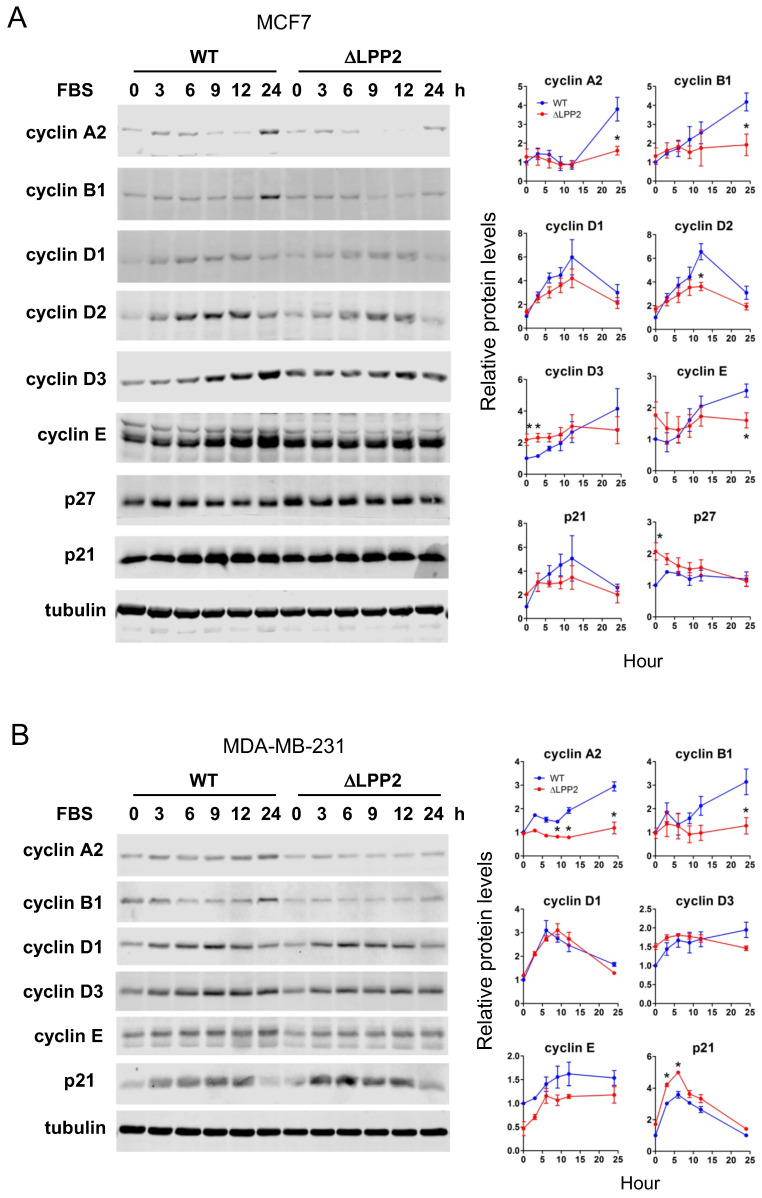
** Effects of LPP2 KO (ΔLPP2) on expression of cell cycle regulators. A:** MCF7 cells were serum starved for 24 h and treated with 10% FBS in culture media. LPP2 KO in MCF7 cells inhibits FBS-induced expression of cyclin A2, cyclin B1, cyclin D2 and cyclin E compared with the wild type (WT) cells. Cyclin D3 and p27 were increased in serum starved cells by LPP2 KO. **B:** MDA-MB- 231 cells were serum starved for 24 h and treated with 10% FBS in culture medium. LPP2 KO in MDA-MB-231 cells inhibits FBS-induced expression of cyclin A2 and cyclin B1 and increases FBS-induced p21 expression compared with the wild-type (WT) cells. Results are means ± SE from three experiments per group and analyzed by ANOVA followed by Tukey test. *P < 0.05 compared with WT.

We tested if the effects of LPP2 KO on expression of cell cycle regulators were in agreement with the accumulation of cells in G1/0. Serum starvation for 24 h maintained low levels of cyclins, p21, and p27. Adding back 10% FBS induced expression of these cell cycle regulators to different extents and with different dynamics. Significant increases of cyclin A2 and B1 were observed in MCF7 cells at 24 h after FBS stimulation (Figure 4A). These changes were suppressed by 61% and 54%, respectively, in LPP2 KO cells. Cyclin D2 levels peaked 12 h after FBS addition and this was decreased by ~38% in LPP2 KO cells (Figure 4A). The levels of cyclin D3 and E remained relatively constant from 3 to 24 h after addition of FBS in LPP2 KO cells. LPP2 KO cells showed significantly higher levels of cyclin D3 levels at 0 and 3 h, and a decrease in cyclin E levels at 24 h relative to wild type cells (Figure 4A). LPP2 KO cells had higher p27 levels at 0 h compared to wild type cells (Figure 4A). Levels of cyclin D1 and p21 did not show significant differences between wild type and LPP2 KO MCF7 cells (Figure 4A).

Similar results were observed in MDA-MB-231 cells, in which LPP2 KO inhibited FBS- induced expression of cyclin A2 and B1, and increased expression of p21 (Figure 4 B).

### LPP2 promotes cell proliferation and increases the expression of cyclins

**Figure 5:**
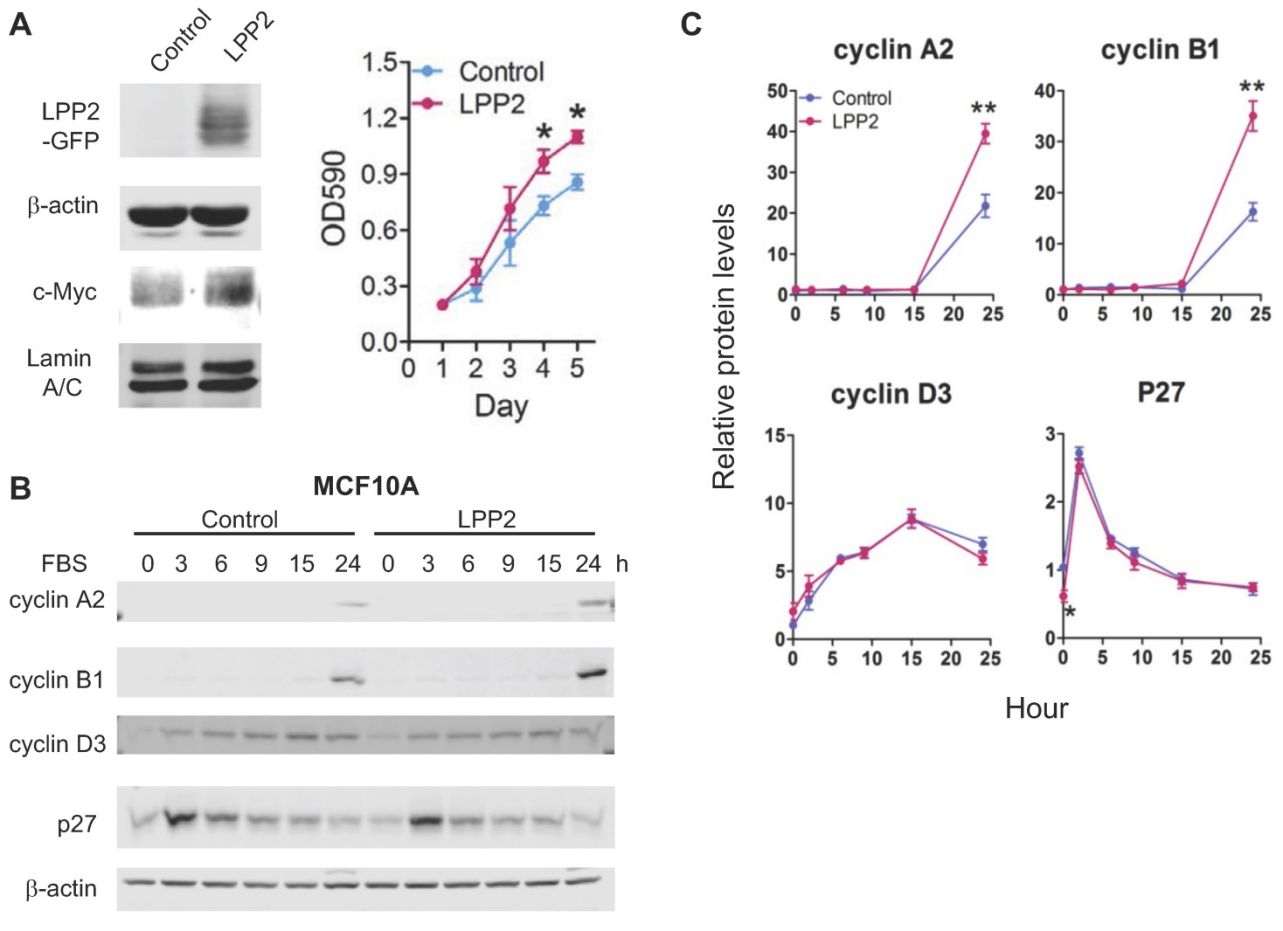
** A:** MCF10A cells expressing GFP-tagged LPP2 (LPP2) showed an increased proliferation and upregulated nuclear c-Myc level compared with cells expressing GFP (Control). **B and C:** MCF10A cells were serum starved for 16 h and treated with 10% FBS in culture media. Cells expressing GFP-tagged LPP2 (LPP2) showed an increase in expression of cyclin A2 and cyclin B1 24 h after stimulation with 10% FBS compared with the cells expressing GFP (Control). After serum starvation, p27 was decreased in cells expressing LPP2 compared with the control. Results are means ± SE from three experiments per group and analyzed by ANOVA followed by Tukey test. *P < 0.05 relative to control.

To increase the level of LPP2, MCF10A cells were transduced with lentivirus to express GFP (control) or GFP-tagged LPP2. Expressing LPP2 significantly increased cell proliferation by ~28% (Figure 5A). The increment of cyclin A2 and cyclin B1 after 24h-stimulation with FBS in MCF10A cells expressing LPP2 were ~81% and ~115% higher than in the control cells. Changes in cyclin D3 were not significantly affected by LPP2 KO. The basal level of p27 in MCF10A cells expressing LPP2 was ~41% lower than the control cells (Figure 5B and C). Similarly, expressing GFP-tagged LPP2 in Hs-578Bst cells also showed a ~40% increase in proliferation relative to the cells expressing GFP (Supplementary Figure 3B).

In agreement with these results, HEK293 and MDA-MB-231 cells expressing GFP-tagged LPP2 have decreased cells of G1/0 phase (Supplementary Figure 3C and D).

### Correlation between LPP2 and c-Myc expression

**Figure 6:**
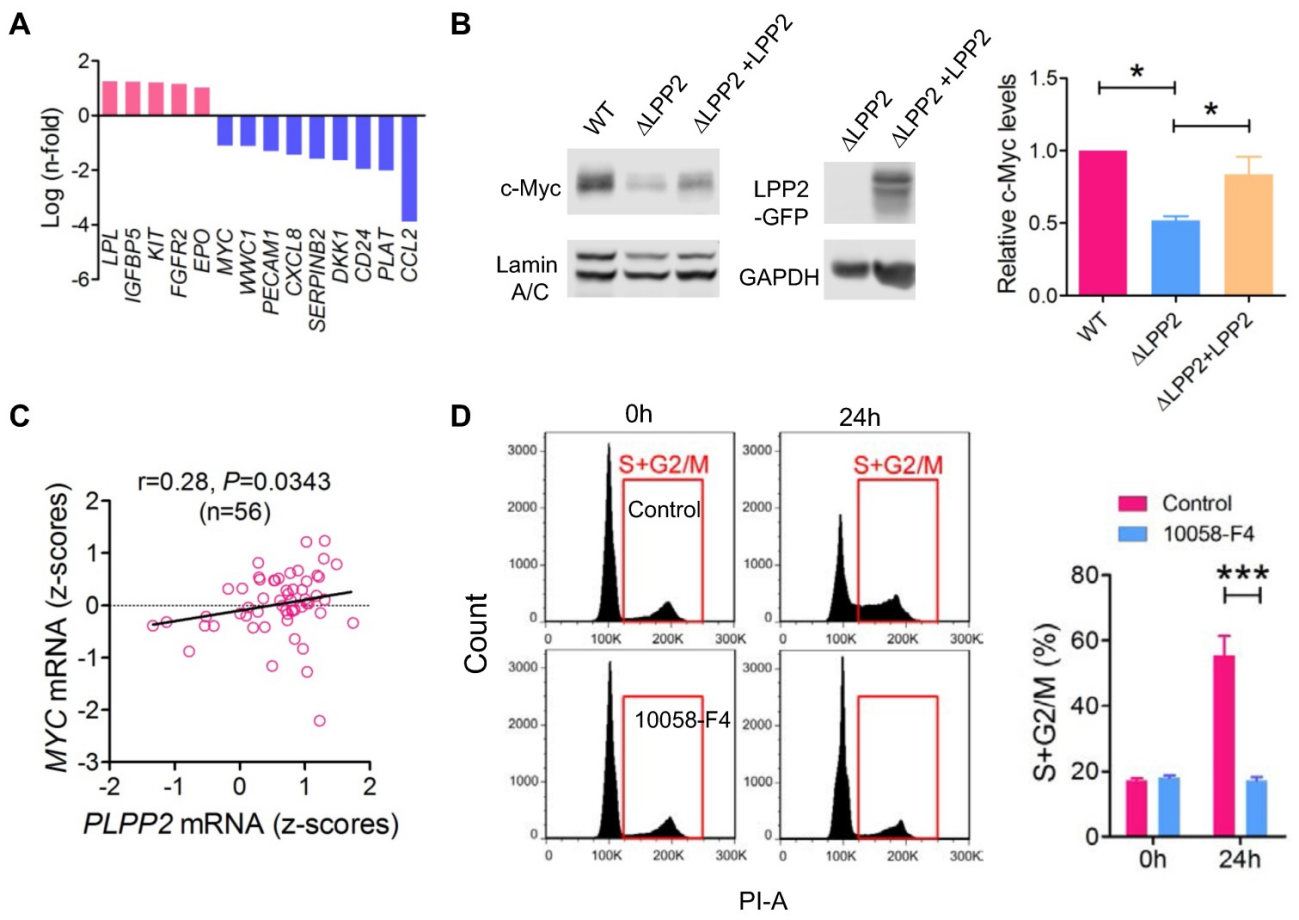
** Cell cycle block caused by LPP2 KO (ΔLPP2) was associated by decrease in c-Myc expression. A:** mRNA with > 2-fold change induced by LPP2 KO in MDA-MB-231 cells. **B:** Decrease of c-Myc by LPP2 KO in the nuclear portion of MDA-MB-231 cells. Re-expression of LPP2 partially restore the c-Myc level in the nuclear. **C:** LPP2 (*PLPP2*) mRNA level is positively correlated with *MYC* mRNA in 56 human breast cancer cell lines. Correlation was determined with Pearson correlation coefficient. **D:** Pretreatment with 50 µM 10058-F4 for 24 h blocked the entry of Lovastatin-synchronazed MDA-MB-231 cells into S and G2/M phase. Results are means ± SE from three experiments per group and analyzed by ANOVA followed by Tukey test. *P < 0.05, ***P < 0.001.

To investigate the cause of the cell cycle inhibition by LPP2 KO, we compared the expression profile of 168 genes relevant to cancer signaling and cancer stem cells in WT and LPP2 KO MDA-MB-231 cells (Table S1). Five genes (*LPL, IGFBP5, KIT, FGFR2,* and* EPO*) showed > 2-fold increased expression, and nine genes (*MYC, WWC1, PECAM1, CXCL8, SERPINB2, DKK1, CD24, PLAT* and *CCL2*) showed > 2-fold decreased expression with LPP2 KO (Figure 6A). CCL2 mRNA levels were decreased by ~16-fold in LPP2 KO cells. We checked the significance of this by measuring cytokines in the conditioned medium of MDA-MB-231 cells. LPP2 KO completely eliminated CCL2 production and also decreased the concentration of GM-CSF. It also increased IL-10 and TNFα concentrations (Supplementary Figure 5C). To test if the elimination of CCL2 accounts for the inhibition in cell cycle progression by LPP2 KO, we treated MDA-MB-231 cells with 100 ng/ml CCL2 for 24 h. This did not induce cell cycle changes in either wild type or LPP2 KO MDA-MB-231 cells (Supplementary Figure 5D), indicating that CCL2 was not responsible for the decrease in cell proliferation.

*MYC*, which is a gene that was down-regulated by LPP2 KO in our array, is an important proto-oncogene that promotes cell cycle progression and is highly expressed in breast tumors. Nuclear c-Myc protein levels were decreased by ~48% in MDA-MB-231 cells with LPP2 KO, and this was partially restored (~66%) by re-expression of LPP2 (Figure 6B). Similarly, LPP2 KO decreased the nuclear expression of total c-Myc and phosphorylated (S62 and T58) c-Myc in HEK293 and MCF-7 cells (Supplementary Figure 6A). Overexpression of GFP-tagged LPP2 in MCF10A cells increased c-Myc level in the nuclear fraction (Figure 5A). We observed a positive correlation between LPP2 (*PLPP2*) and *MYC* mRNA expression in 56 breast cancer cell lines (Figure 6C) through analyzing the CCLE database. This positive correlation also exists in human lung, upper aerodigestive tract, and urinary tract cancer cell lines (Supplementary Figure 6B). LPP1 and LPP3 mRNA levels did not correlate with *MYC* mRNA levels (results not shown). C-Myc inhibitor 10058-F4 mimicked the LPP2 KO-induced blockade of entry into S phase in lovastatin-synchronized MDA-MB-231 cells (Figure 6D).

### LPP2 knockout suppresses breast tumor growth *in vivo*

**Figure 7:**
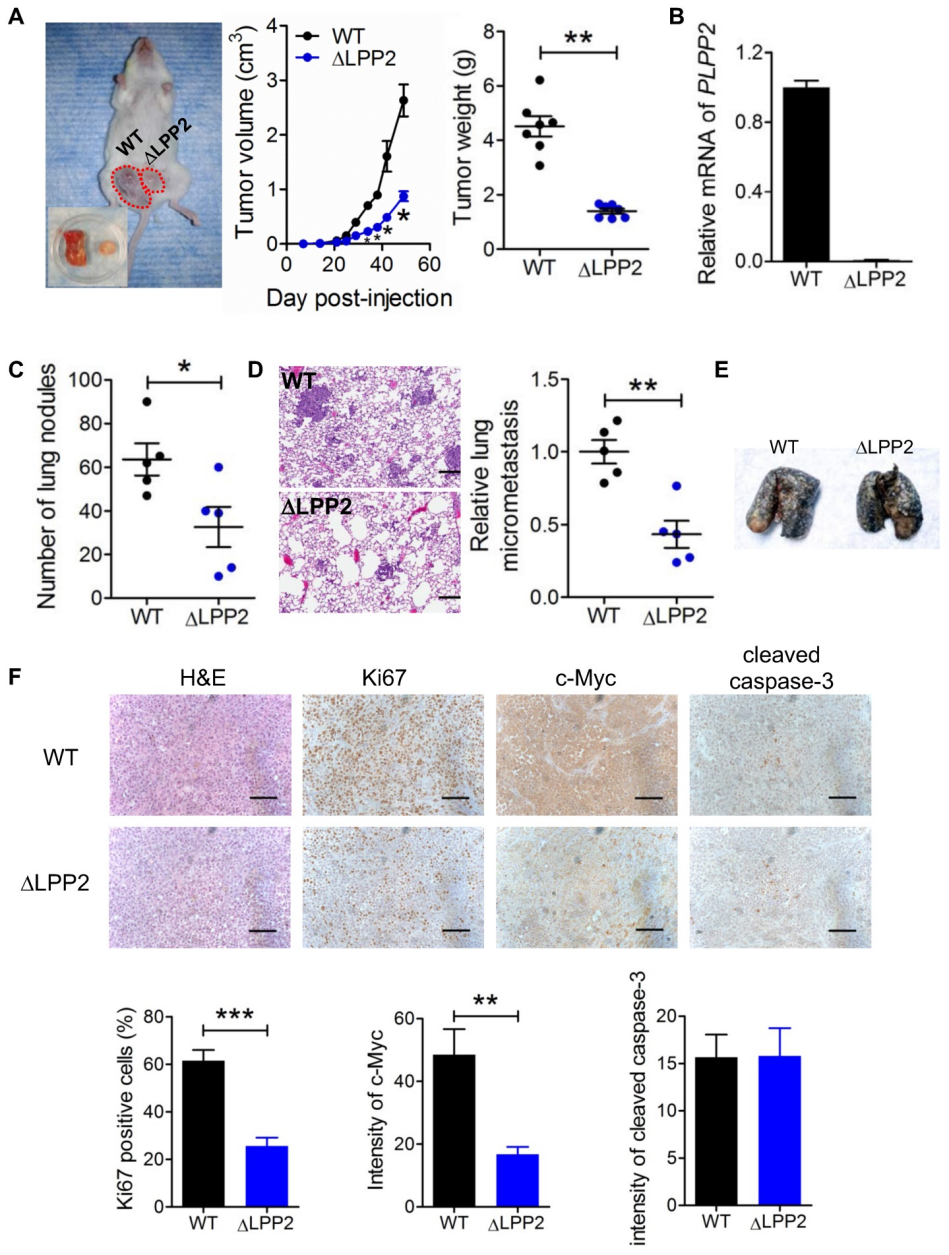
** LPP2 KO (ΔLPP2) in MDA-MB-231 breast cancer cells decreased tumor growth. A:** Tumor volume and tumor weight from MDA-MB-231 cells in a xenograft mouse model of breast cancer. The image is a representative of two mice with both wild type (WT, left) and LPP2 KO (right) tumors. Another ten mice had single tumors, five for WT and five for LPP2 KO. **B:** Human LPP2 was not detectable by qRT-PCR in tumors formed by LPP2 KO MDA-MB-231 cells. **C:** MDA-MB-231 cells with LPP2 KO formed less visible nodules on lungs. **D:** MDA-MB-231 cells with LPP2 KO formed less micro metastasis in lungs. **E:** Representative image of lungs from the experimental lung metastasis model established by tail vein injection of WT or LPP2 KO MDA-MB-231 cells. Lungs were perfused with Indian ink. The white spots on the surface of the lungs are nodules of tumor. **F:** Immunohistochemistry staining and quantification of Ki67, c-Myc, and cleaved caspase 3 in the tumors. Scale bar = 100 µm. Results were means ± SE from n=5 or 7 per group. Results were analyzed by two-tail t-test or ANOVA followed by Tukey test. * *P* < 0.05, **P < 0.01, ***P < 0.001 compared with WT.

We next assessed the consequences of LPP2 KO on breast tumor growth *in vivo* by injecting human MDA-MB-231 cells into the mammary fat pads of NSG mice. LPP2 KO decreased tumor volumes and weights by ~67% and ~68%, respectively, compared to control grafts (Figure 7A).

We verified LPP2 knockout using the primers targeting the cleavage in exon 3 of human LPP2 gene. No PCR product was detected from the tumors formed by LPP2 KO cells (Figure 7B). Visible nodules on lung and micro-metastasis were decreased by ~49% (Figure 7C) and ~57% (Figure 7D) with LPP2 KO, respectively. We also established an experimental lung metastasis model by injecting MDA-MB-231 cells through the tail vein. We did not observe any differences in the number of visible lung nodules between wild type and LPP2 KO (Figure 7E), suggesting that the decrease in spontaneous lung metastasis by LPP2 KO probably resulted from the lower mass of the primary tumor. In the tumors, LPP2 KO decreased the percentage of Ki67 positive cells and c-Myc expression in cancer cells by ~58% and ~65% respectively, compared with the tumors developed from wild type cells (Figure 7F). Cleaved caspase-3 in tumors was not affected by LPP2 KO (Figure 7F).

We also explored the effects of LPP2 KO in a syngeneic mouse model with 4T1 mouse breast cancer cells. LPP2 KO in 4T1 cells caused a ~53% decrease in LPP activity (Supplementary Figure 7A) and a downregulation of nuclear c-Myc levels (Supplementary Figure 7B). Tumor volumes and weights were decreased by ~40% and ~35% respectively with LPP2 KO (Supplementary Figure 7C). The number of visible nodules on lungs was not significantly changed by LPP2 KO (Supplementary Figure 7D).

## Discussion

In the present work, we showed that LPP2 KO in breast cancer cells delays the transition from G_1_ to S-phase of the cell cycle. This explains why knockout of LPP2 in breast cancer cells decreases tumor growth in two different mouse models. Part of this effect can be explained by the discovery that nuclear c-Myc is increased by LPP2 expression and decreased in response to LPP2 KO in several cell lines. This is supported by the positive correlation between LPP2 and *MYC* mRNA levels found in 56 human breast cancer cell lines and also in human lung, upper aerodigestive tract, and urinary tract cancer cell lines. *MYC* constitutes a family of proto-oncogenes including *c-MYC*, *MYCN* and *MYCL* in mammalian cells 31. c-Myc regulates cell cycle, protein synthesis, cell adhesion, and metabolism through targeting as many as 15% of all genes by forming heterodimers with Max or Miz-1 32. As a rate-limiting step in G1/S transition in cancer cells 33-36, c-Myc accelerates the cell cycle and enhances proliferation 31. Reducing c-Myc levels causes growth arrest not only in *MYC*-driven cancers, but also in cancers driven by other oncogenes 37. This is because c-Myc activates the transcription of cyclins, cyclin-dependent protein kinase (CDK), and represses the cell cycle inhibitors p21 and p27 32, 38-40. The effect on c-Myc is specific to LPP2, since there is no significant correlation between LPP1 and LPP3 and *MYC* mRNA levels (results not shown). We did not observe a significant correlation between LPP2 and *MYC* in human breast tumors (results not shown). This is probably because of the heterogeneity of tumor tissue, which contains around 50% of stromal cells 41.

KIT and FGFR2 are receptor tyrosine kinases which were upregulated by LPP2 KO in the PCR array analysis. KIT is a proto-oncogene and KIT mutations are associated with several human malignancies 42. However, KIT mutation is rare in breast cancer 43 and loss of KIT expression occurs during mammary transformation 44, 45. FGFR2 mediates signaling from fibroblast growth factors. Its function in breast cancer is also controversial and not clearly understood 46. CXCL8 mRNA was downregulated by LPP2 KO in the PCR array analysis, however, the protein concentration of CXCL8 (IL-8) in the conditioned medium was not significantly changed. LPP2 KO in MDA-MB-231 cells completely eliminated the expression of CCL2, a chemokine that attracts macrophages and regulates cancer cell migration and survival 47, 48. However, treatment with exogenous CCL2 did not affect the cells in G1/0 phase, ruling out an effect of CCL2 on cell cycle. IL-10 and TNFα concentrations in the conditioned media of MDA-MB-231 cells were significantly increased by LPP2 KO. Although high dose of TNFα induces apoptosis of cancer cells 49, 50, increases in TNFα in the tumor microenvironment is considered as a stimulator of pro-tumorigenic inflammation 51, 52. IL-10 can act as an anti-inflammatory cytokine. Administration of exogenous IL-10 inhibits tumor growth by activating CD8^+^ T cells and interferon-γ secretion 53, 54, which also showed better outcome when in combination with PD-1 blockade 55. On the other hand, blocking IL-10 signaling also has an antitumor effect 56, which is synergistically enhanced by immune checkpoint inhibition 57. This reflects the complexity of IL-10 function in cancers. Changes of cell cycle regulators caused by LPP2 KO in this study are consistent with the phenotype of c-Myc deficiency 58, and therefore is probably due to the reduction of c-Myc.

*MYC* amplification is implicated in ~50% of human cancers 59, 60, and high expression of c-Myc is associated with a worse prognosis in breast cancer as indicated by a recent Meta-Analysis 61. c-Myc is highly activated in triple negative breast tumors 62, suggesting its potential role as a therapeutic target. Unfortunately, c-Myc itself is not an easily “druggable” protein because of the lack of a specific active site or a deep pocket on its protein surface suitable for small-molecule binding 37, 63. Despite of issues with target selectivity, rapid metabolism and low potency, current small molecular inhibitors for c-Myc have shown promising effects that inhibit tumor growth in animal models 64. Our study provides a possible alternative strategy for inhibiting c-Myc indirectly through decreasing LPP2. The regions composing the active sites of LPP2 have been clarified and these can be used for designing small molecular inhibitors. In addition to regulating cancer cell proliferation and invasion, c-Myc also affects the tumor microenvironment through inducing expression of inflammatory cytokines (IL-1β) and immune checkpoints (CD47 and PD-L1) in cancer cells 65, 66. This is expected to be improved by targeting c-Myc or LPP2.

The ecto-activity of LPP1 and LPP3 against LPA has been shown to be critical for the anti-tumor effects 10, since LPA is a potent mediators of tumor growth, metastasis and inflammation 5-7. LPP2 is also expressed on the plasma membrane and the intracellular membrane system and thus contributes to hydrolyzing extracellular LPA 67. The discrepancies among the actions of LPP2 versus LPP1 and LPP3 could be caused by differences in their intracellular locations and the different substrates that are dephosphorylated by LPP2 and LPP1/3 *in vivo*.

The present experiments demonstrate for the first time that knockout LPP2 in breast cancer cells decreases tumor growth in mouse models of breast cancer. This is partly mediated by decreasing the expression of c-Myc. The present work adds to the limited body of knowledge that has been assembled to define the signaling properties of LPP2. In addition, a major contribution of the present work is to explain why high expression of LPP2 in various tumors is associated with a poor prognosis for disease-free survival in cancer patients. This study provides “proof of principle” that counteracting high LPP2 expression in cancer cells could represent a novel strategy for decreasing tumor growth by attenuation of c-Myc signaling.

## Supplementary Material

Supplementary figures and table.

## Abbreviations

BrdU: bromodeoxyuridine
CCL2: C-C motif chemokine ligand 2
CRISPR: clustered regularly interspaced short palindromic repeats
DMEM: Dulbecco's modified eagle medium
EGF: epidermal growth factor
ER: estrogen receptor
ERK: Extracellular signal-regulated kinase
FBS: fetal bovine serum
GAPDH: glyceraldehyde 3-phosphate dehydrogenase
GM-CSF: granulocyte-macrophage colony-stimulating factor
HER2: human epidermal growth factor receptor 2
IGF: insulin-like growth factor
IL: interleukin
LPA: lysophosphatidate
LPP: lipid phosphate phosphatase
PAR1: protease-activated receptor-1
PARP: poly(ADP-ribose) polymerase
PI: propidium iodide
PR: progesterone receptor
S1P: sphingosine 1-phosphate
TNFα: tumor necrosis factor α
WT: wild type
