# Supplementary material for: Lipid phosphate phosphatase-2 promotes tumor growth through increased c-Myc expression
Source: Theranostics. 2022 Jul 18;12(13):5675–90. doi: 10.7150/thno.66230 (PMC9373824; doi:10.7150/thno.66230)
Supplement: Supplementary file 1 — Supplementary figures and table. [file thnov12p5675s1.pdf]

Supplementary Figure 1

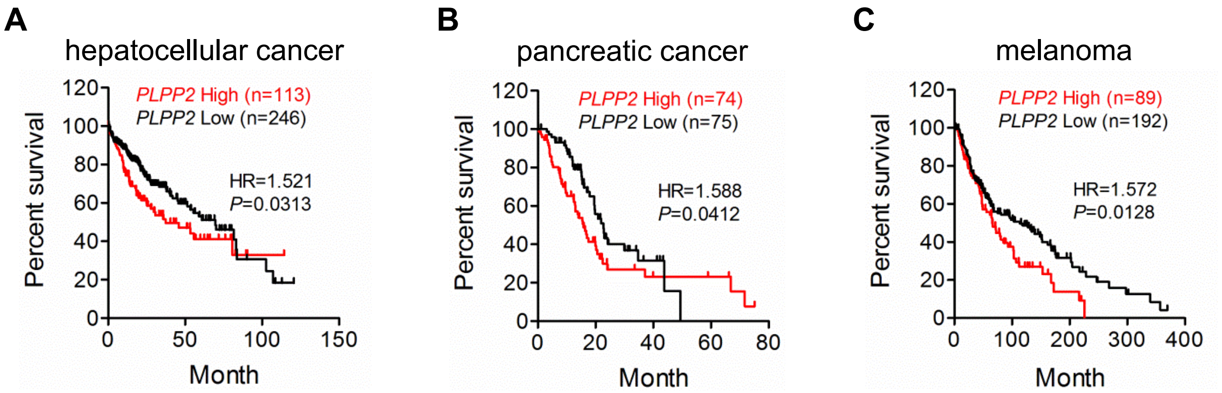

Supplementary Figure 1: A-C: Disease-free survival curves from patients with high (Z score > 0) and low (Z score < 0) mRNA levels of LPP2 (*PLPP2*) were plotted using data extracted from TCGA database. Results were analyzed by log rank test.

Supplementary Figure 2

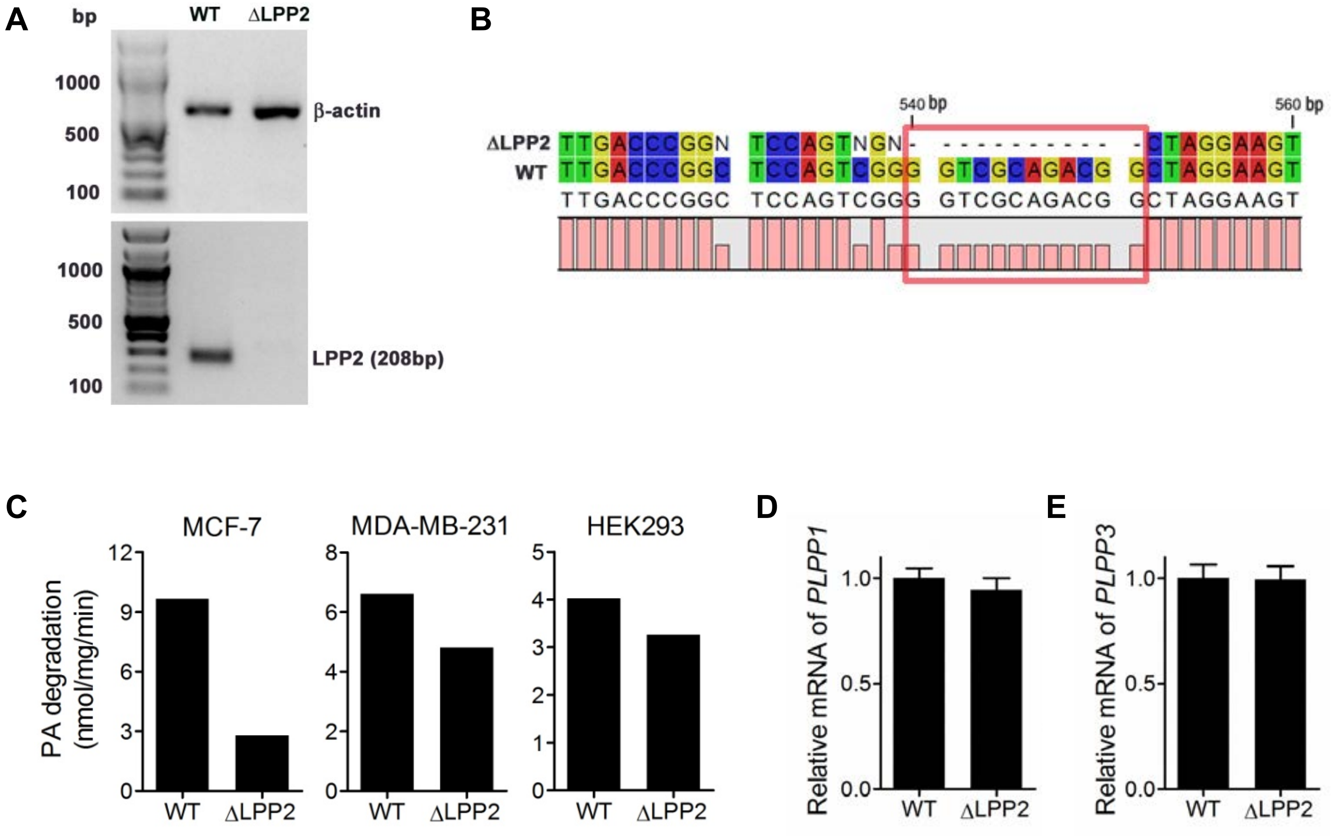

Supplemeraty Figure 2: Confirmation of LPP2 knockout. **A:** PCR of LPP2 using cDNA of wild type (WT) and LPP2 knockout (ΔLPP2) MCF7 cells. One of the primers is complimentary with the Cas9 cleavage site in exon 3 of human LPP2 gene. The predicted size of the amplification product is 208 bp. LPP2 knockout cells did not have the PCR product because the sequence at the Cas9 cleavage site has been disrupted by the indels. **B:** The indel (marked with red box) at the Cas9 cleavage site of human LPP2 gene was confirmed by genomic sequencing. **C:** LPP activity decreased by 75%, 27%, and 19% in MCF7, MDA-MB-231, and HEK293 cells by LPP2 knockout . The remaining LPP2 activity is due to other LPP isoforms e.g. LPP1 and LPP3. **D and E:** Levels of LPP1 and LPP3 mRNA in MCF7 cells were not affected by LPP2 knockout.

Supplementary Figure 3

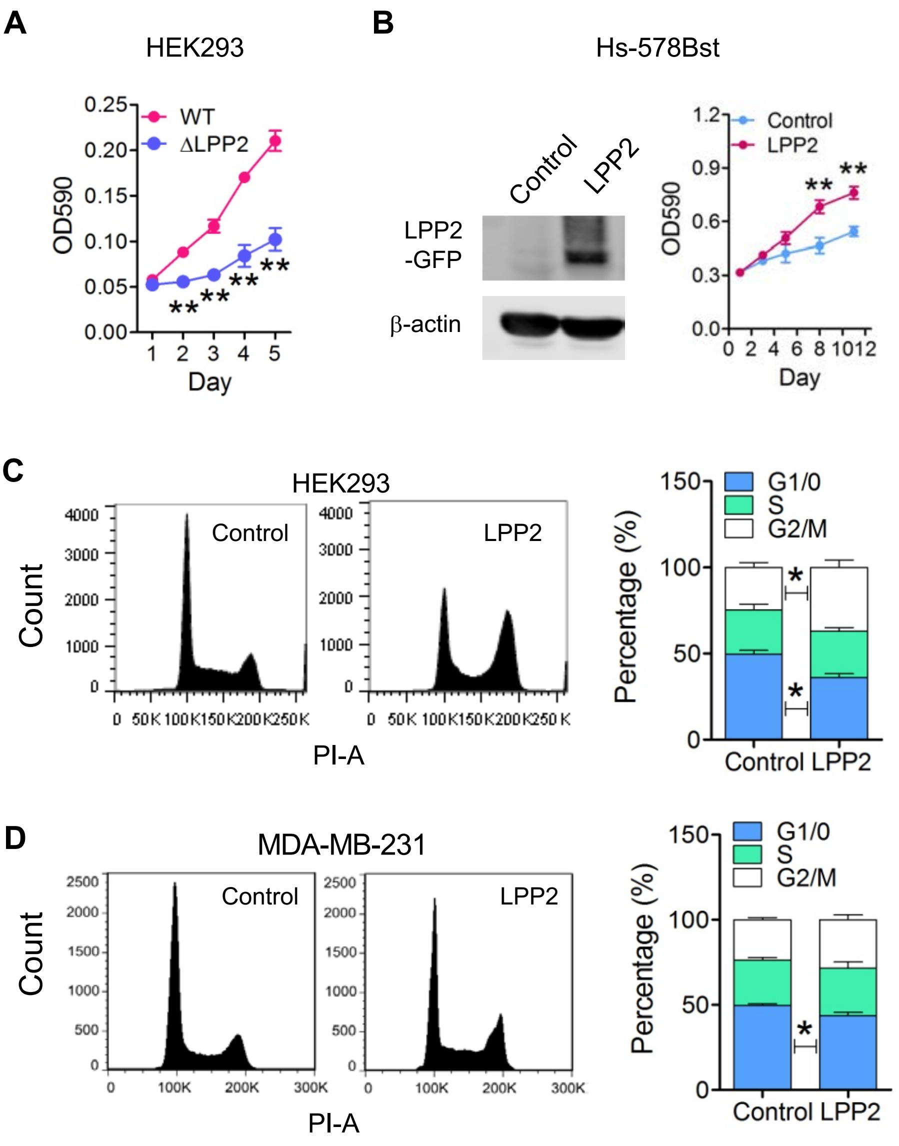

Supplemeraty Figure 3: A: Knockout of LPP2 (ΔLPP2) inhibited proliferation of HEK293 cells. **B:** Hs-578Bst cells expressing GFP-tagged LPP2 (LPP2) showed an increased proliferation compared with cells expressing GFP (Control). **C and D:** Expressing GFP-tagged LPP2 in HEK293 and MDA-MB-231 cells decreased the percentage of cells in G1/0 phase. Results are means ± SE from three experiments per group and analyzed by ANOVA followed by Tukey test. *$P < 0.05$, **$P < 0.01$ compared with control.

Supplementary Figure 4

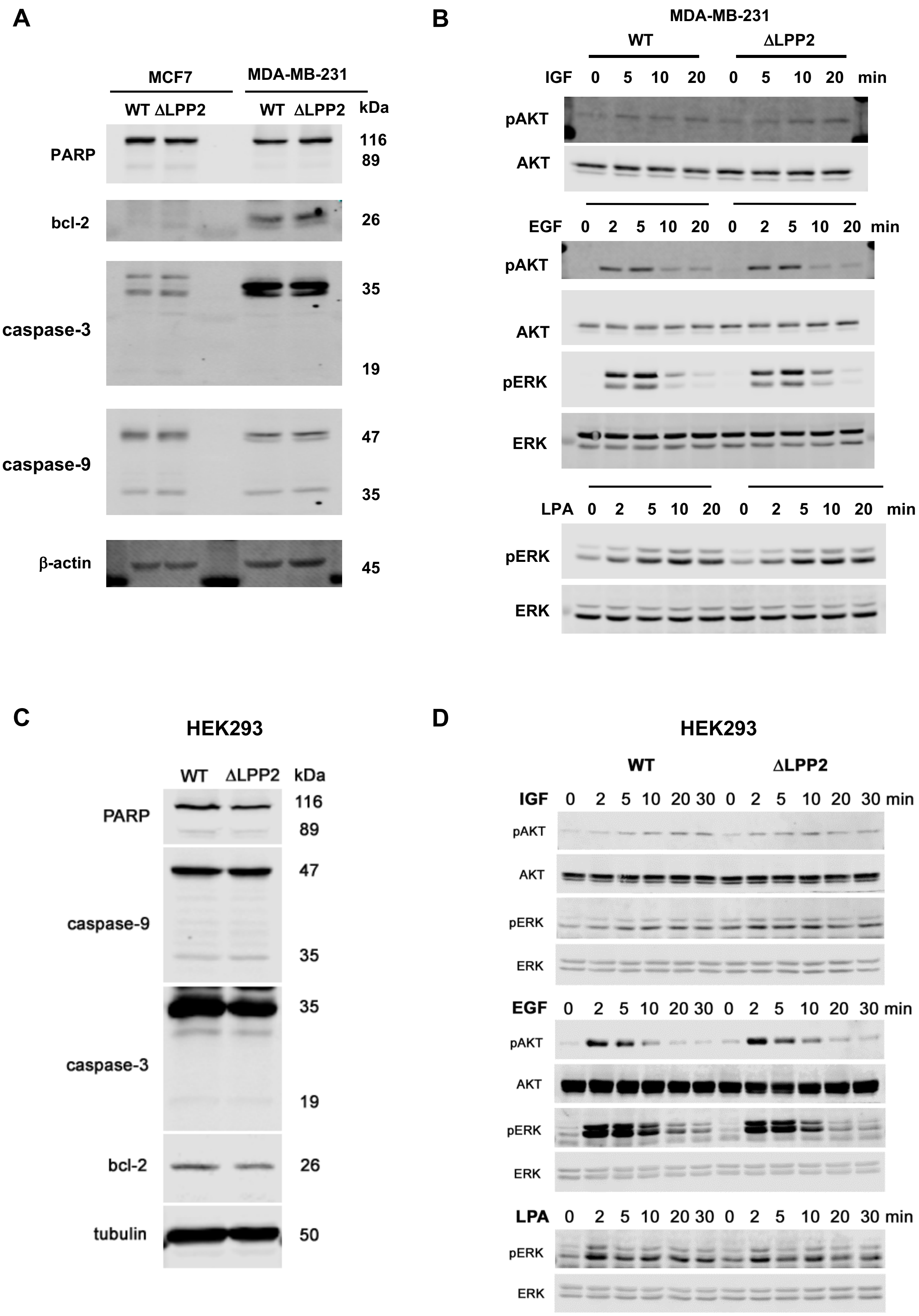

Supplemeraty Figure 4: A: Expressions of PARP, caspase-9, caspase-3 and bcl-2 in wild type (WT) and LPP2 knockout (ΔLPP2) MCF7 and MDA-MB-231 cells. **B:** Time courses of AKT and ERK phosphorylation induced by IGF (50 ng/ml), EGF (50 ng/ml) and LPA (10 μM) in wild type (WT) and LPP2 knockout (ΔLPP2) MDA-MB-231 cells. **C:** Expressions of PARP, caspase-9, caspase-3 and bcl-2 in wild type (WT) and LPP2 knockout (ΔLPP2) HEK293 cells. **D:** Time courses of AKT and ERK phosphorylation induced by IGF (50 ng/ml), EGF (50 ng/ml) and LPA (10 μM) in wild type (WT) and LPP2 knockout (ΔLPP2) HEK293 cells.

Supplementary Figure 5

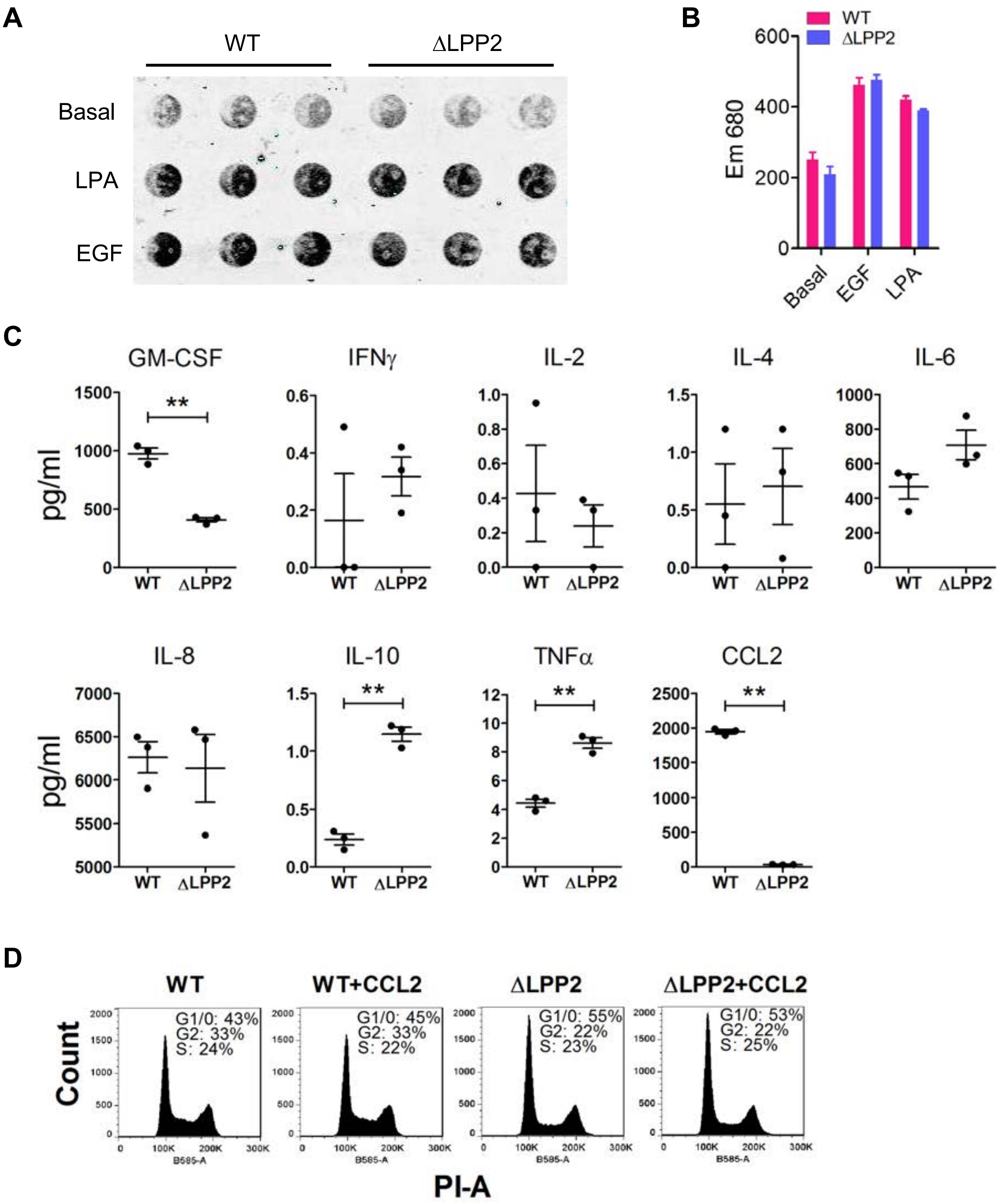

Supplementary Figure 4: A : Wild type (WT) and LPP2 knockout (ΔLPP2) MDA-MB-231 cells migrated through a 8μm-pore filter in a Boyden chamber assay were fixed with methanol. The round bands of cells were stained with crystal violet. **B:** The intensity of cell bands were quantified by LI-COR infra-red imaging system. **C:** The decrease of CCL2 mRNA expression was proved by measuring cytokines in the conditioned media of MDA-MB-231 cells. Secretion of CCL2 was almost completely suppressed by LPP2 knockout. LPP2 knockout also significantly inhibit the secretions of GM-CSF and increased IL-10 and TNFα. **D:** Wild type (WT) and LPP2 knockout (ΔLPP2) MDA-MB-231 cells treated with 100 ng/ml CCL2 for 24 h did not show changes in cell cycle relative to cells without CCL2 treatment. Results were analyzed by two-tail t-test or ANOVA followed by Tukey test. $^{**}P < 0.01$ compared with WT.

Supplementary Figure 6

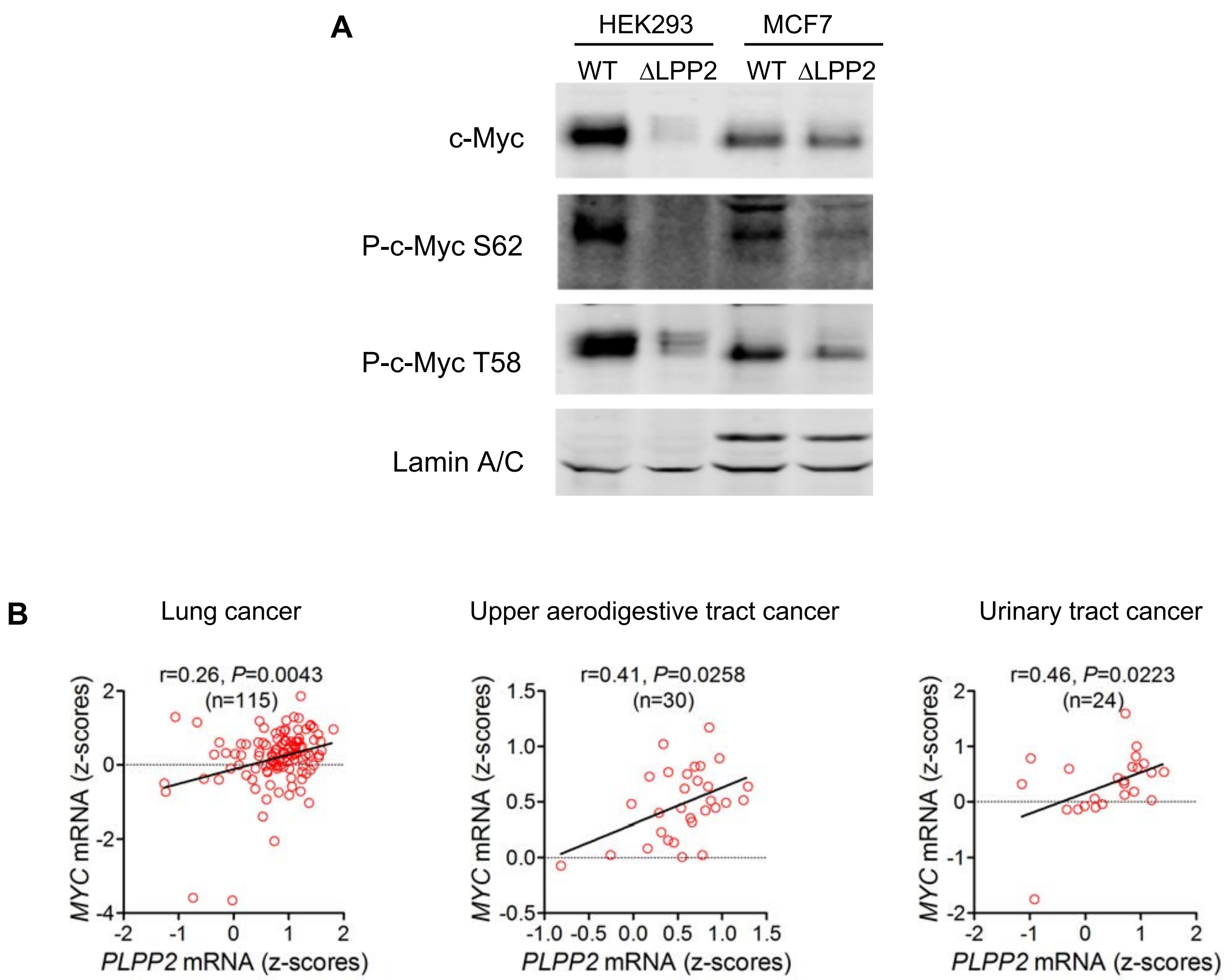

Supplementary Figure 6: A: Decrease of c-Myc by LPP2 KO (ΔLPP2) in nuclear portions of HEK293 and MCF7 cells. Phospho-c-Myc (S62 and T58) in LPP2 KO cells also decreased compared with the wild type (WT) cells. **B:** Positive correlation between LPP2 (*PLPP2*) and c-Myc (*MYC*) mRNA in cell lines of lung, upper aerodigestive tract, and urinary tract cancer. Data were extracted from Cancer Cell Line Encyclopedia (CCLE) of the Broad Institute and Novartis and Pearson correlation coefficient was determined.

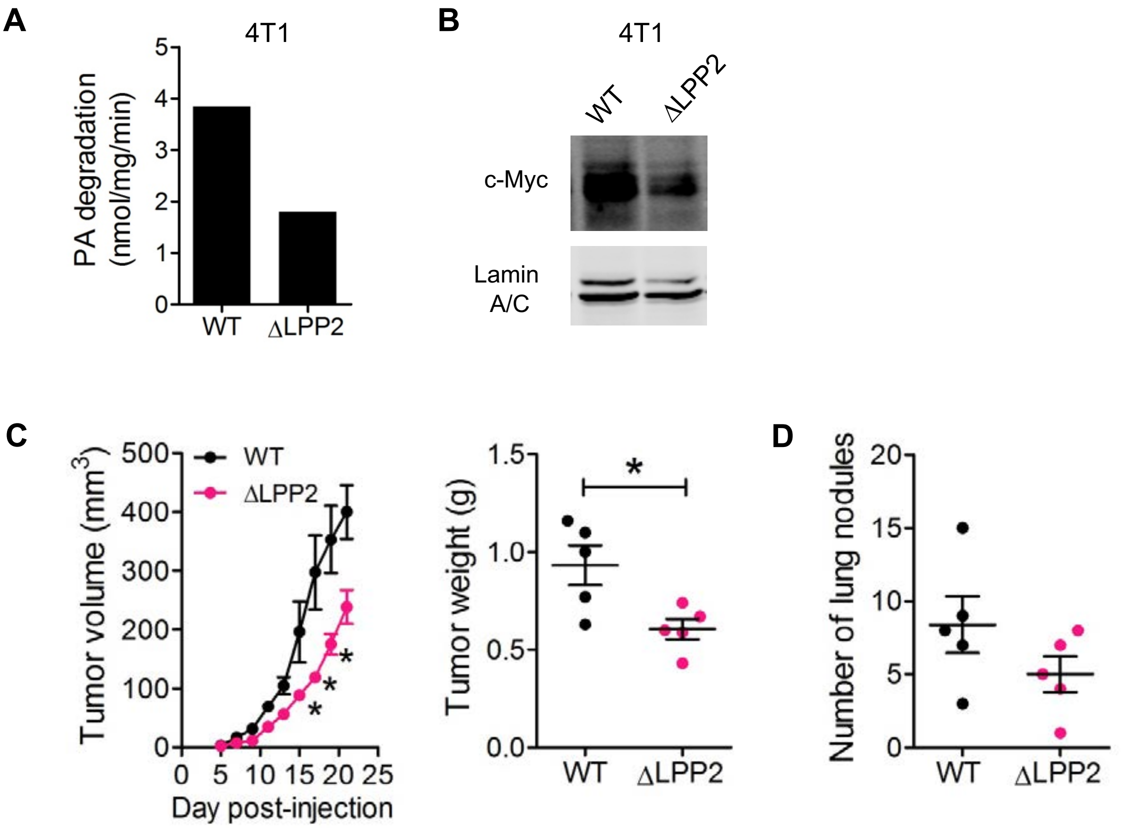

Supplementary Figure 7: A: LPP activity decreased by ~53% in 4T1 mouse breast cancer cells by LPP2 knockout (ΔLPP2) . **B:** Decrease of c-Myc by LPP2 KO in nuclear portions of 4T1 cells. **C:** LPP2 KO in 4T1 cells decreased tumor growth and tumor weight in a syngeneic mouse model of breast cancer. **D:** LPP2 KO in 4T1 cells decreased number of visible nodules on lungs. Results were means ± SE from n = 5 per group. Results were analyzed by two-tail t-test or ANOVA followed by Tukey test. * $P < 0.05$ compared with WT.

Supplementary Table 1

Supplementary table 1: changes of gene expression in LPP2 knockout MDA-MB-231 cells relative to wild-type cells

up-regulated genes in LPP2 knockout MDA-MB-231 cells

Symbol	Genbank	log2 (n-fold) changes
LPL	NM_000237	1.254634857
IGFBP5	NM_000599	1.241355896
KIT	NM_000222	1.212060928
FGFR2	NM_000141	1.152915955
EPO	NM_000799	1.019714355
HMOX1	NM_002133	0.999263763
TERF1	NM_017489	0.992427826
SERPINF1	NM_002615	0.93470192
PROM1	NM_006017	0.9311409
ALDH1A1	NM_000689	0.879224777
TWIST2	NM_057179	0.87663269
CDH2	NM_001792	0.858652115
FASLG	NM_000639	0.856519699
ITGA2	NM_002203	0.845849991
WNT1	NM_005430	0.843910217
ANGPT2	NM_001147	0.819452286
TEK	NM_000459	0.814302444
FGF2	NM_002006	0.812753677
POU5F1	NM_002701	0.787111282
SMO	NM_005631	0.769031525
LIN28B	NM_001004317	0.768035889
LIN28A	NM_024674	0.756601334
THY1	NM_006288	0.712741852
DLL1	NM_005618	0.6901474
CD38	NM_001775	0.688327789
BIRC3	NM_001165	0.664369583
SNAI3	NM_178310	0.661207199
ANGPT1	NM_001146	0.651576996
FOXP1	NM_032682	0.624868393
ITGA4	NM_000885	0.595752716
ID1	NM_002165	0.589834213
GADD45G	NM_006705	0.589595795
SOX10	NM_006941	0.589097977
SOX2	NM_003106	0.561719894
SNAI2	NM_003068	0.547794342
NOS2	NM_000625	0.5260849
CCND2	NM_001759	0.522829056
PTPRC	NM_002838	0.518320084
ENG	NM_000118	0.518217087
CD34	NM_001773	0.438417435
FLT1	NM_002019	0.424293518
KLF17	NM_173484	0.41472435
MS4A1	NM_021950	0.413740158
ATXN1	NM_000332	0.406332016
KRT14	NM_000526	0.403690338
XIAP	NM_001167	0.393486023
TWIST1	NM_000474	0.391210556
KDR	NM_002253	0.378587723
NANOG	NM_024865	0.353549957
PGF	NM_002632	0.34327507
ZEB1	NM_030751	0.330713272
CA9	NM_001216	0.324865341
G6PD	NM_000402	0.314731598
GSC	NM_173849	0.298522949
ABCB5	NM_178559	0.289064407
APAF1	NM_001160	0.272527695
BMP7	NM_001719	0.272285461
DLL4	NM_019074	0.262916565

down-regulated genes in LPP2 knockout MDA-MB-231 cells

Symbol	Genbank	log2 (n-fold) changes
CASP7	NM_001227	-0.002742767
MAP2K1	NM_002755	-0.003427505
ADM	NM_001124	-0.014028549
IGFBP7	NM_001553	-0.018899918
PTCH1	NM_000264	-0.05238533
ATM	NM_000051	-0.05688858
GSK3B	NM_002093	-0.073875427
ZEB2	NM_014795	-0.097764969
TAZ	NM_000116	-0.118747711
IKBKB	NM_001556	-0.154550552
TERF2IP	NM_018975	-0.156988144
YAP1	NM_006106	-0.187217712
TGFBR1	NM_004612	-0.206331253
PFKL	NM_002626	-0.207624435
ITGB1	NM_002211	-0.220739365
DDIT3	NM_004083	-0.222034454
FZD7	NM_003507	-0.223829269
MAML1	NM_014757	-0.228210449
LATS1	NM_004690	-0.233034134
ABCG2	NM_004827	-0.243246078
COX5A	NM_004255	-0.24379158
CASP9	NM_001229	-0.244665146
CD44	NM_000610	-0.288244247
CASP2	NM_032982	-0.290599823
NOTCH2	NM_024408	-0.291160583
ACLY	NM_001096	-0.299726486
JAG1	NM_000214	-0.302106857
ALCAM	NM_001627	-0.303314209
NOTCH1	NM_017617	-0.303665161
FOXC2	NM_005251	-0.313022614
WEE1	NM_003390	-0.323949814
E2F4	NM_001950	-0.324035645
GPD2	NM_000408	-0.326490402
HDAC1	NM_004964	-0.326719284
CCND3	NM_001760	-0.337615967
TEP1	NM_007110	-0.35941124
ERCC5	NM_000123	-0.364494324
MKI67	NM_002417	-0.364543915
KITLG	NM_003994	-0.367141724
MUC1	NM_001018016	-0.367591858
PINX1	NM_017884	-0.3705616
SOD1	NM_000454	-0.397607803
CPT2	NM_000098	-0.398511887
MAP2K3	NM_002756	-0.410093307
OCLN	NM_002538	-0.421339035
DDB2	NM_000107	-0.421834946
PLAUR	NM_002659	-0.458185196
VEGFC	NM_005429	-0.461988449
JAK2	NM_004972	-0.466907501
STMN1	NM_005563	-0.470554352
WEE1	NM_003390	-0.472980499
TINF2	NM_012461	-0.480195999
GATA3	NM_002051	-0.48400116
ATP5A1	NM_004046	-0.484085083
SKP2	NM_005983	-0.485609055
PPP1R15A	NM_014330	-0.512840271
ETFA	NM_000126	-0.521837234
AURKA	NM_003600	-0.535881042

Supplementary table 1: changes of gene expression in LPP2 knockout MDA-MB-231 cells relative to wild-type cells

up-regulated genes in LPP2 knockout MDA-MB-231 cells

Symbol	Genbank	log2 (n-fold) changes
SNAI1	NM_005985	0.260686874
TNKS	NM_003747	0.248916626
DACH1	NM_004392	0.219028473
TBX2	NM_005994	0.217012405
DDR1	NM_001954	0.199293137
IGFBP3	NM_000598	0.159706116
BCL2L11	NM_006538	0.14899826
LDHA	NM_005566	0.13892746
MYCN	NM_005378	0.127464294
TNKS2	NM_025235	0.100011826
MAPK14	NM_001315	0.095556259
EGF	NM_001963	0.042631149
MERTK	NM_006343	0.036523819
ARNT	NM_001668	0.031070709
NOL3	NM_003946	0.021102905

down-regulated genes in LPP2 knockout MDA-MB-231 cells

Symbol	Genbank	log2 (n-fold) changes
UQCRFS1	NM_006003	-0.537349701
DNMT1	NM_001379	-0.545078278
CFLAR	NM_003879	-0.546897888
FLOT2	NM_004475	-0.548576355
SIRT1	NM_012238	-0.554803848
AXL	NM_001699	-0.577075958
SAV1	NM_021818	-0.588003159
POLB	NM_002690	-0.592323303
ETS2	NM_005239	-0.600931168
ERCC3	NM_000122	-0.611265182
SLC2A1	NM_006516	-0.661832809
CHEK1	NM_001274	-0.682130814
LIG4	NM_002312	-0.688751221
ERBB2	NM_004448	-0.696914673
NFKB1	NM_003998	-0.69950676
DKC1	NM_001363	-0.702711105
BMI1	NM_005180	-0.72023201
STAT3	NM_003150	-0.72908783
FOXA2	NM_021784	-0.735729218
ACSL4	NM_004458	-0.754804611
ITGA6	NM_000210	-0.756126404
MCM2	NM_004526	-0.76002121
DSP	NM_004415	-0.784818649
CDC20	NM_001255	-0.866754532
KLF4	NM_004235	-0.940618515
EPCAM	NM_002354	-0.951040268
MYC	NM_002467	-1.110683441
WWC1	NM_015238	-1.114034653
PECAM1	NM_000442	-1.303861618
CXCL8	NM_000584	-1.436033249
SERPINB2	NM_002575	-1.590080261
DKK1	NM_012242	-1.640377045
CD24	NM_013230	-1.961645126
PLAT	NM_000930	-2.01742363
CCL2	NM_002982	-3.887592316
